# A computational DNA methylation method to remove contaminated DNA from spent embryo culture medium for noninvasive preimplantation genetic testing

**DOI:** 10.1016/j.ebiom.2025.105669

**Published:** 2025-03-29

**Authors:** Yidong Chen, Jin Huang, Fuchou Tang, Lu Wen, Jie Qiao

**Affiliations:** a Biomedical Pioneering Innovation Center, State Key Laboratory of Female Fertility Promotion, Center for Reproductive Medicine, Department of Obstetrics and Gynecology, Third Hospital, School of Life Sciences, Peking University, Beijing, China; b Beijing Advanced Innovation Center for Genomics, Third Hospital, Peking University, Beijing, China; c Key Laboratory of Assisted Reproduction and Key Laboratory of Cell Proliferation and Differentiation, Ministry of Education, Beijing, China; d Beijing Key Laboratory of Reproductive Endocrinology and Assisted Reproductive Technology, Beijing, China; e Peking-Tsinghua Center for Life Sciences, Academy for Advanced Interdisciplinary Studies, Peking University, Beijing, China

**Keywords:** Spent embryo culture medium, DNA methylation, Computational method, Noninvasive preimplantation genetic testing

## Abstract

**Background:**

In the last fifty years, assisted reproductive technology (ART) has achieved remarkable breakthroughs, culminating in the birth of 12 million infants. At the heart of ART success is preimplantation genetic testing (PGT), which enables the detection of chromosomal anomalies, single-gene disorders, and structural rearrangements, enhancing embryo selection and mitigating genetic risk. However, current PGT methods, including trophectoderm (TE) biopsy, face limitations such as challenges related to convenience and potential impacts on embryonic health. In this evolving field, noninvasive PGT (niPGT) has emerged as a promising alternative. By analysing cell-free DNA (cfDNA) in spent embryo culture medium (SECM), niPGT offers a less intrusive approach. However, maternal DNA contamination within SECM remains a marked barrier to its clinical application as underscored by our research and other studies. There is an urgent need for innovation and optimisation in niPGT methodologies.

**Methods:**

We developed a computational algorithm to eliminate contaminated nonembryonic DNA from spent embryo culture medium. The rationale is based on the phenomenon that the DNA methylation level of a mammalian preimplantation embryo reaches its minimum at the blastocyst stage during a global DNA demethylation wave. Therefore, selecting hypomethylated reads is expected to enrich blastocyst DNA over nonembryonic DNA. To investigate this, we retrieved single-cell-resolution DNA methylation data from oocytes (n = 33), inner cell masses (ICMs, n = 74), TEs (n = 71) and sperm cells (n = 21), bulk DNA methylation data from cumulus cells, and DNA methylation data from SECM samples (n = 194) from our previously published database, and conducted a comparative analysis of DNA methylation patterns among them. Then, we constructed a decontamination algorithm based on single read and applied it to remove contamination originating from cumulus cells, polar bodies, and sperm cells.

**Findings:**

By selecting hypomethylated reads, we successfully enriched blastocyst DNA over DNA originating from cumulus cells, polar bodies and sperm (enrichment factors = 4, 1.2, and 2.5, respectively). By testing simulated SECM samples, the method demonstrated a substantial reduction in the false-negative rate even with up to 75% cumulus cell contamination. In real clinical SECM samples, the method improved aneuploidy detection sensitivity at a cumulus cell contamination ratio of 50%.

**Interpretation:**

Our study introduces a novel computational strategy for reducing nonembryonic DNA contamination, thereby enhancing aneuploidy detection sensitivity in SECM cfDNA methylation analyses. In combination with DNA methylation methodologies, this approach holds considerable promise for advancing niPGT applications in ART.

**Funding:**

This study was supported by grants from the Beijing Natural Science Foundation (7232203), the 10.13039/501100012166National Key R&D Program of China (2023YFC2705600, 2023YFC2705602), the 10.13039/501100001809National Natural Science Foundation of China (82301889, 82371706), the Key Clinical Projects of 10.13039/501100009399Peking University Third Hospital (BYSYZD2022029), the Young Elite Scientists Sponsorship Program by CAST (2023QNRC001), the Peking University Medicine Sailing Program for Young Scholars’ Scientific & Technological Innovation (BMU2023YFJHPY001) and the special fund of the National Clinical Key Specialty Construction Program, P. R. China (2023). We thank support from the High Performance Computing Platform of the 10.13039/501100011620Centre for Life Sciences (10.13039/501100007937Peking University) and Open Research Fund of the National Centre for Protein Sciences at 10.13039/501100007937Peking University in Beijing.

**Research in context**

**Evidence before this study**

Noninvasive preimplantation genetic testing for aneuploidies (niPGT-A) is a promising advancement in reproductive medicine but faces challenges such as maternal DNA contamination in SECM, which compromises chromosome ploidy accuracy. In our previous work using whole-genome DNA methylation analysis, we identified cfDNA in SECM as originating from blastocyst, cumulus cells, and polar bodies. Notably, two-thirds of SECMs presented with maternal contamination above 20%, resulting in sex discordances and false negatives. Current strategies to reduce contamination focus primarily on refining sample collection but remain labour intensive, only partially effective, and increase in vitro fertilization (IVF) lab workloads. This highlights the urgent need for simpler, more efficient decontamination methods.

**Added value of this study**

For the first time, we present a computational method to effectively remove contaminated DNA from SECM cfDNA, enhancing the enrichment of blastocyst DNA over that from cumulus cells, polar bodies, and sperm DNA. This decontamination algorithm is straightforward and powerful, integrating seamlessly into existing bioinformatics workflows for analysing DNA methylation data from SECM samples. This approach offers a feasible solution to the issue of DNA contamination in SECM, thus paving the way for advanced noninvasive embryo screening and establishing a robust foundation for advancements in reproductive medicine.

**Implications of all the available evidence**

Our research reveals that the hypomethylated read segment is instrumental in effectively enriching blastocyst DNA in SECM, eliminating contaminated DNA, and increasing the sensitivity of aneuploid detection. These findings suggest that analogous strategies could be applied to the detection of single-gene disorders and structural rearrangements, further expanding the horizons of genetic diagnostics in reproductive medicine.

## Introduction

Preimplantation genetic testing for aneuploidies (PGT-A) has demonstrated clinical utility of assessing chromosome abnormalities in human embryos, yet it remains a subject of ongoing debate and extensive research.^1^ Some studies have suggested that PGT-A could improve pregnancy and implantation rates, whereas others have questioned its overall clinical benefits.^2^, ^3^, ^4^ A comprehensive review of 11 randomised trials indicates that PGT-A provides benefits in terms of live birth rates, at least for women over 35 years of age undergoing fertility treatment.^5^ Some negative aspects of PGT-A may result from the potential invasiveness associated with trophectoderm (TE) biopsy.

Noninvasive preimplantation genetic testing of aneuploidy (niPGT-A), which examines the cell-free DNA (cfDNA) of spent embryo culture medium (SECM) for PGT-A, is emerging as a promising alternative approach to TE biopsy.^6^, ^7^, ^8^, ^9^, ^10^ NiPGT-A does not affect the embryo itself in principle and is more convenient than TE biopsy.^11^^,^^12^ Compared with standard PGT-A, it may offer greater sensitivity and reliability.^13^ A large-scale multicentre prospective study involving 1301 human blastocysts revealed high consistency (78.2%) between SECM cfDNA and TE biopsy.^14^

However, one challenge of niPGT-A is the presence of maternal contamination in SECM, which compromises the accuracy of chromosome ploidy detection. Vera-Rodriguez et al. showed that in SECM, maternal DNA contamination was high (86–94%), and the median proportion of embryonic DNA was approximately 8% according to Single Nucleotide Polymorphism (SNP) analysis.^15^ Rubio et al. demonstrated the existence of maternal contamination in SECM by recognising sex inconsistency between TE biopsy and SECM.^16^ Unlike these studies, which used genomic information, we previously investigated the cellular origin of cfDNA in SECM via whole-genome DNA methylation analysis and reported that the cfDNA in SECM was derived from blastocyst, cumulus cells and polar bodies. We showed that approximately two-thirds of SECMs had a net maternal DNA contamination ratio greater than 20%, which can lead to sex differences and false negative results.^10^^,^^17^

**Fig. 1:**
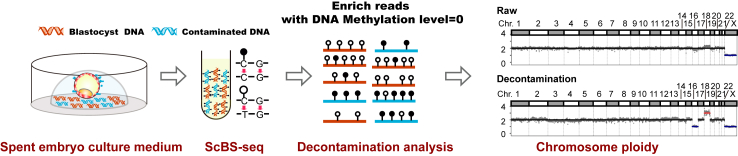
**Study outline.** The SECM, which contains cell-free DNA from the blastocyst and may be contaminated with other sources, is collected and subjected to PBAT-based single-cell whole-genome DNA methylation sequencing (scBS-seq) for DNA methylation analysis. A computational decontamination algorithm reduces contaminated nonembryonic DNA by selecting read segments with DNA methylation levels of 0. Subsequently, the embryonic CNV profile is identified. The black circle represents methylated cytosine, and the white circle represents unmethylated cytosine. After bisulfite treatment, the black circle represents cytosine, and the white circle represents thymine.

In this study, we developed a novel approach to reduce maternal contamination in SECM computationally by using DNA methylation information. This innovative approach capitalises on a critical epigenetic event: the global demethylation wave that occurs during the preimplantation phase of mammalian development, resulting in the lowest methylation levels at the blastocyst stage.^18^ Leveraging this phenomenon, we propose a strategy to selectively enrich blastocyst DNA by identifying and prioritising hypomethylated reads, thereby differentiating it from the DNA of maternal cumulus cells. We showed that, by selecting unmethylated reads, blastocyst DNA can be enriched over maternal cumulus cell DNA, and this bioinformatics process can reduce false negative rates (FNR) in aneuploid SECM samples with maternal contamination. This strategy aims to increase the diagnostic accuracy of niPGT-A (Fig. 1).

## Methods

### Data source

All the sequencing data used in this study were obtained from our team's previous research,^10^ and the data have been deposited in the National Genomics Data Centre (https://bigd.big.ac.cn/gsa/), with accession number HRA000332. A total of 194 PGT-A blastocysts and their corresponding culture media were included in this study. In all of these PGT-A cycles, the mode of fertilisation is via intracytoplasmic sperm injection (ICSI) on the day of egg retrieval.

### Ethics

This study was approved by the Reproductive Medicine Ethics Committee of Peking University Third Hospital (Research Licence 2019-393-02). All participants provided written informed consent.

### Data processing

We performed data processing as described in our previously published article.^10^^,^^17^ In brief, first, sequencing adaptors, amplification primers, and low-quality bases in the raw sulfite sequencing reads were removed. Then, R2 reads with more than 3 unmethylated CHs were discarded along with the corresponding R1 reads. BS-Seeker2 (v2.1.1) was used to map the clean reads to the human reference genome (hg19) first in end-to-end alignment mode and then in local alignment mode. Next, PCR duplicates were removed via the Picard tool (v1.119). The DNA methylation level was calculated as the ratio of the number of methylated C reads to the total number of reads. Only CpG sites covered by more than three reads were retained for calculation. Samples with more than 1 million uniquely mapped reads were retained for further analysis.

### DNA methylation signature analysis of cumulus cell and SECM reads

MethylDackel (v0.6.1) perRead^19^ was employed to assess CpG methylation levels in cumulus cells and SECM samples by counting methylated (mC) and total cytosines (C) per read, computing the methylation ratio (mC/C), and providing statistical methylation insights. Then, the methylation level condition with the most enriched embryonic cell DNA was screened, the read fragment data were retained under this condition, and the chromosome copy number of the embryo was calculated.

### Simulation analysis of different proportions of cumulus/sperm/polar body contamination samples

We first selected high-quality non-contaminated aneuploid SECM samples and TE and ICM samples and then calculated the number of unique mapping reads. For example, the SECM sample had 1,000,000 unique mapping reads. We aimed to synthesise a mixed sample with 50% cumulus cells (sperm cells/MII oocytes), and then, we randomly collected 1,000,000 unique mapping reads from all cumulus cells (sperm cells/MII oocytes) and mixed into a new simulated sample.

### Inferring copy number variation (CNV) profiles

We used the ginkgo (http://qb.cshl.edu/ginkgo)^20^ with some modifications to evaluate chromosome copy number variations. First, we divided the entire genome into 2705 variable-length bins with a median length of 1 Mb and excluded aberrant bins that were blacklisted by Ginkgo. Browser Extensible Data (BED) files converted from aligned Binary Alignment/Map (BAM) files via bedtools (v2.22.1) were used as input files. Lowess normalisation was used to correct for genomic Guanine-Cytosine (GC) content bias. BED files synthesised from randomly extracted normal diploid reads were used as references. The CNV threshold can be adjusted through the fixed[,k]∗CN in the R script named 'process' that comes with the Ginkgo software. CNV line plots are drawn in ggplot2.

$$\text{GCR}=\frac{\text{SECMeuploid}\;\times \;\text{TEeuploid}\;+\;\text{SECManeuploid}\;\times \;\text{TEaneuploid}}{\text{all}\;\text{SECM}}$$

The General Concordance Rate (GCR) is calculated as the sum of the concordant euploid and aneuploid embryos from both SECM and TE, divided by all SECM samples. GCR stands for the general concordance rate between the ploidy results of the SECM and TE. It refers to the ratio of samples that exhibit either euploidy or aneuploidy in both TE and SECM, relative to the overall sample count.

$$\text{FNR}=\frac{\text{SECMeuploid}\;\times \;\text{TEaneuploid}}{\text{SECMeuploid}\;\times \;\text{TEaneuploid}\;+\;\text{SECManeuploid}\;\times \;\text{TEaneuploid}}$$

FNR is calculated by dividing the number of samples identified as euploid by SECM but aneuploid by TE biopsy by the number of samples identified as euploid by SECM but aneuploid by TE biopsy, plus the number of samples identified as aneuploid by both SECM and TE biopsy. FNR refers to the proportion of actual positives that are incorrectly classified as negatives. It should be noted that this calculation assumes the TE result to be the gold standard, which is not always the case due to embryonic mosaicism. One critical cause of false negatives is maternal contamination, which leads to samples being incorrectly identified as euploid in SECM but actually aneuploid according to TE. In such cases, an embryo might be transferred due to the erroneous classification, increasing the risk of miscarriage. Additionally, false negatives can arise from embryonic mosaicism that leads to a discordance between SECM and TE.

$$\text{FPR}=\frac{\text{SECManeuploid}\;\times \;\text{TEeuploid}}{\text{SECManeuploid}\;\times \;\text{TEeuploid}\;+\;\text{SECMeuploid}\;\times \;\text{TEeuploid}}$$

The False Positive Rate (FPR) is calculated by dividing the number of samples identified as aneuploid by SECM but euploid by TE biopsy by the number of samples identified as aneuploid by SECM but euploid by TE biopsy, plus the number of samples identified as euploid by both SECM and TE biopsy. FPR refers to the proportion of actual negatives that are incorrectly classified as positives. False positives can occur due to embryonic mosaicism or technical artifacts in the SECM examination. In such cases, an embryo with reproductive potential may be mistakenly discarded.

$$\text{SDR}=\frac{\text{SECMfemale}\;\times \;\text{TEmale}}{\text{SECMfemale}\;\times \;\text{TEmale}\;+\;\text{SECMmale}\;\times \;\text{TEmale}}$$

The Sex Discordance Rate (SDR) is calculated by dividing the number of samples identified as female by SECM but male by TE biopsy by the number of samples identified as female by SECM but male by TE biopsy, plus the number of samples identified as male by both SECM and TE biopsy. SDR stands for sex discordance rate. Specifically, due to maternal contamination, samples identified as male embryos from TE results are incorrectly diagnosed as female embryos by SECM.

### Decontamination analysis of various samples

The number of CpG sites and the average methylation level of each read in a sample were calculated via MethylDackel perRead.^19^ Next, reads with CpG numbers greater than 1 and average methylation levels equal to 0 were retained. The reads that met the above conditions from the corresponding BED files were subsequently extracted, and the Copy Number (CN) variations were calculated.

### Statistics

Statistical analysis was performed in R (v4.1.0) via tests as stated in the figure legends. A t-test was performed for each reporting threshold group to assess statistical significance compared to the decontamination alone group in Supplementary Fig. S5. A p value less than 0.05 was considered statistically significant.

### Role of funders

The sources of funding did not influence the design of the study, the collection of data, the analysis of the data, the interpretation of the results, or the writing of the manuscript.

## Results

### Enriching blastocyst DNA by selecting hypomethylated reads

First, we investigated whether DNA from blastocyst (including the inner cell mass (ICM) and TE) and SECM without cumulus cell contamination can be distinguished from DNA from cumulus cells at the single-read level. From our previously published database,^10^^,^^18^ we retrieved single-cell-resolution DNA methylation data of the ICM and TE, bulk DNA methylation data of cumulus cells, and DNA methylation data of SECM samples (n = 96) that had minor cumulus cell contamination (<20%) and presented the highest concordance in CNV with the corresponding TE biopsy results.^10^ All the data were obtained via the post bisulfite adaptor tagging (PBAT) method, and thus the comparability was high.^21^

On average, the whole-genome methylation levels of the ICM, TE, and SECM samples and cumulus cells were 24%, 24%, 29% and 71%, respectively. In each dataset, approximately 70% of the reads contained at least one CpG site, which provided information for read-level methylation analysis. On average, 24%, 15%, 9%, and 19% of the reads contained one, two, three or more than three CpGs, respectively (Supplementary Fig. S1).

**Fig. 2:**
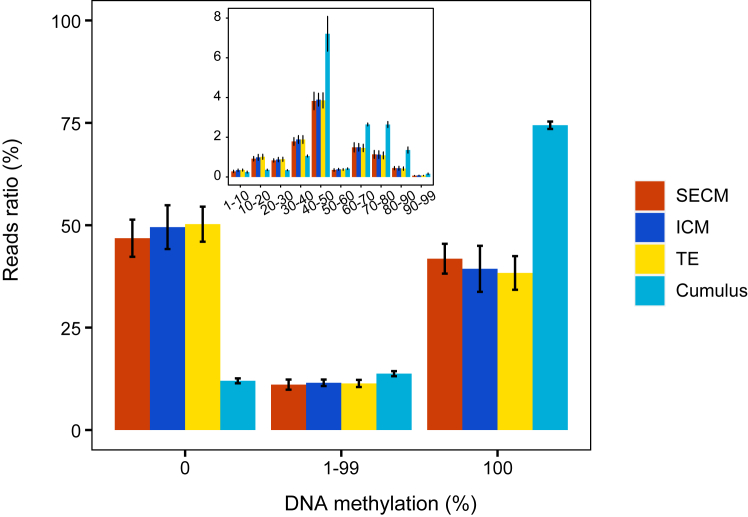
**Characterisation of the methylation distribution in SECM, ICM, TE and cumulus cells.** The X-axis represents the average DNA methylation level on each read segment. The Y-axis represents the ratio of read segments under the corresponding conditions. Among them, SECMs are completely uncontaminated culture medium samples.

We calculated the percentage value of methylated CpGs to all CpGs on each read. For the SECMs, ICM and TE, on average, 47%, 50%, and 50% of all the CpG-containing reads presented a methylation value of 0, respectively; 11%, 12%, and 11% presented values between 1% and 99%, respectively; and 42%, 39% and 38% presented values of 100%, respectively. The cumulus cells presented a distinct pattern from those of the SECMs, ICM and TE. Among all the CpG-containing reads, 12%, 14% and 74% had average values of 0%, 1–99%, and 100%, respectively (Fig. 2). If the unmethylated reads with a methylation value of 0% are selected, the blastocyst DNA is expected to be enriched over the cumulus cell DNA by a factor of approximately 4 [47% divided by 12%, where 47% and 12% refer to the average ratios of the reads with a CpG methylation value of 0 in the blastocyst-representative samples (which include the SECM with minor contamination, ICM and TE) and cumulus cell samples, respectively]. If the selection included hypomethylated reads with a methylation value between 1% and 99%, the read number increased, but the enrichment factor decreased to approximately 2 (58% divided by 26%). Thus, we selected unmethylated reads for the decontamination operation.

### Decontamination analysis for simulated samples with cumulus contamination

**Fig. 3:**
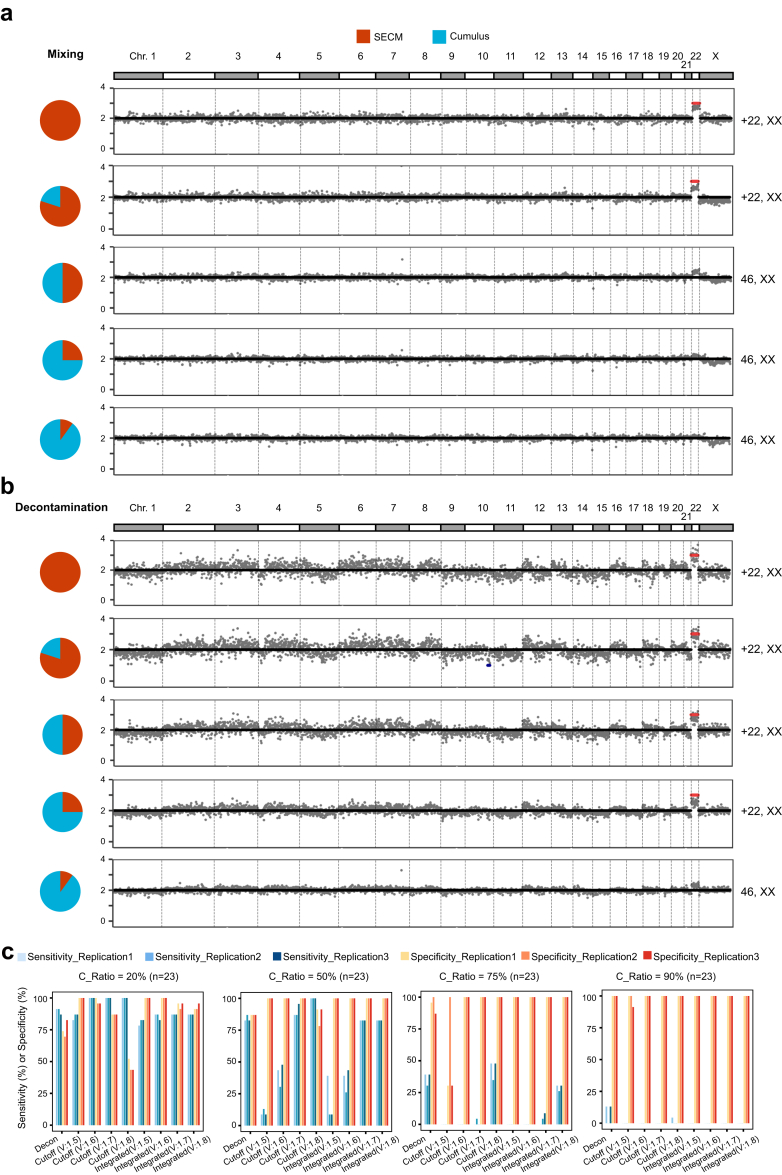
**CNVs of simulated SECMs with different proportions of cumulus cells before and after decontamination.** (a) Raw CNVs of simulated samples with different proportions of cumulus cells. The proportions of cumulus cells were 20%, 50%, 75% and 90%, respectively. (b) CNV of simulated samples with different proportions of cumulus cells after decontamination. The proportions of cumulus cells were 20%, 50%, 75% and 90%, respectively. Among them, SECMs are completely uncontaminated culture medium samples. (c) Sensitivity and specificity under different reporting thresholds in different cumulus cell contamination samples.

Next, we explored whether chromosome CNVs of the blastocyst DNAs can be recovered via SECM with varying degrees of cumulus cell contamination by enriching the blastocyst DNA with unmethylated reads. We synthesised in silico DNA methylation data by mixing the reads of an aneuploid SECM sample (+22, XX, with no maternal contamination) with the reads of cumulus cells at four different ratios, i.e., 20%, 50%, 75%, and 90%. As expected, the signal of the aneuploid chromosome gradually decreased with increasing cumulus cell contamination ratio; aneuploidy was not detected when the proportion of cumulus cell contamination was 50% or higher (Fig. 3a). We then selected the unmethylated reads and performed aneuploidy analysis. The percentage of unmethylated reads were 32%, 25%, 18%, and 12% in the data with cumulus ratios of 20%, 50%, 75%, and 90%, respectively. Notably, the number of aneuploid chromosomes clearly increased in the data with cumulus ratios of 50%, 75% and 90%, and aneuploid chromosomes were recognised in the data with cumulus ratios of 50% and 75% (Fig. 3b). A simulation using another aneuploid SECM sample (−16, +18, XY, with no maternal contamination) yielded similar results, and the improvement was also most prominent for the data with cumulus proportions of 50% and 75% (Supplementary Fig. S2).

We then selected all full-aneuploidy SECM samples free from maternal contamination (n = 23) for simulation analysis. The results revealed that while the FNR was consistently reduced, some samples showed CNVs that was not the original aneuploidy (Supplementary Figs. S3 and S4). In addition, the application of decontamination treatment to real clinical SECM samples with maternal contamination also resulted in a high FPR. We hypothesised that these false positives may be caused by a reduction in the number of read segments during decontamination analysis or a regional imbalance in DNA methylation, introducing noise and fluctuations in CNV detection. To address the high FPR, we considered that a genuine CNV, when obscured by cumulus cell contamination, should manifest as a mosaic-like CNV prior to decontamination. Consequently, we implemented a new algorithm requiring both the presence of a mosaic CNV above a certain threshold before decontamination and a full CNV after decontamination.

We evaluated various predecontamination CNV thresholds, specifically 1.5&2.5 (abbreviated for threshold = 1.5 for a CN loss and threshold = 2.5 for a CN gain), 1.6&2.4, 1.7&2.3 and 1.8&2.2; the post-contamination threshold was consistently set at 1.5&2.5. We generated DNA methylation data for SECMs with varying proportions of cumulus cell contamination and assessed the sensitivity and specificity for detecting CNVs via these different predecontamination CNV thresholds. Each computational experiment was repeated three times.

The results suggested that incorporating predecontamination CNV thresholds improved the specificity; as the threshold increased from 1.8&2.2 to 1.5&2.5, the sensitivity decreased. At a 50% contamination ratio, the specificity increased from 87% (20/23) in the decontamination group alone to 100% (23/23) in all groups with predecontamination threshold integration (Fig. 3c, Supplementary Fig. S5, p-value = 0.047, paired t-test). Moreover, the sensitivity was maintained at 83% (19/23) in the groups with predecontamination thresholds of 1.7&2.3 and 1.8&2.2, respectively, compared with the decontamination group alone. At a 75% contamination ratio, the sensitivity of the group with a predecontamination threshold of 1.8&2.2 matched that of decontamination alone, at approximately 30% (7/23), whereas the sensitivity of the group with a predecontamination threshold of 1.7&2.3 decreased to near zero (0/23).

Therefore, the results demonstrated that integrating a predecontamination CNV threshold of 1.8&2.2 can enhance specificity while preserving similar sensitivity to decontamination alone.

### Decontamination analysis for clinical SECM samples with cumulus contamination

Next, we assessed the efficacy of the algorithms in real clinical SECM samples with maternal contamination. We and others previously reported that SECM samples often contain maternally contaminated DNA.^22^, ^23^, ^24^ Among all 182 SECM samples examined in our previous DNA methylation study, 35% (63/182) were indicated to have a cumulus contamination rate higher than 25%. As we have shown, the FNR increased with increasing maternal contamination ratio; the FNR was 17% (11/65, 95% CI: 8%–26%), 50% (7/14, 95% CI: 24%–76%), 100% (7/7, 95% CI: 90%–100%), and 85% (11/13, 95% CI: 65%–100%) at maternal contamination ratios of less than 25%, between 25% and 50%, between 50% and 75%, and more than 75%, respectively. In contrast, the FPR decreased as the maternal contamination ratio increased, with rates of 39% (21/54, 95% CI: 26%–52%), 40% (2/5, 95% CI: 0%–83%), 17% (2/12, 95% CI: 0%–38%) and 25% (3/12, 95% CI: 0%–49%) corresponding to the same contamination ratio ranges (Supplementary Fig. S6a).

**Fig. 4:**
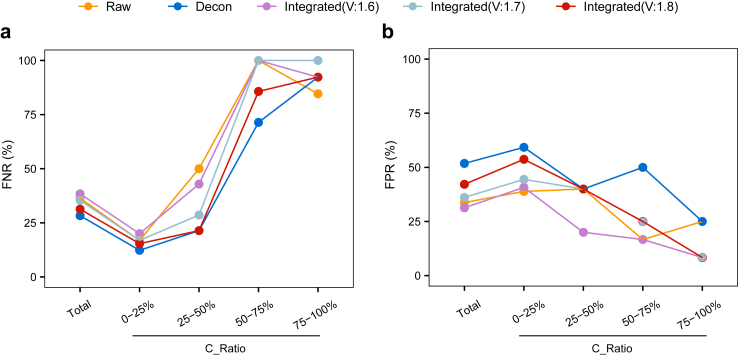
**Performance effects of decontamination strategies on real SECM samples.** Line charts showing the false negative rates (a) and false positive rates (b) under different decontamination strategies.

We applied the decontamination algorithm to the SECM DNA methylation data. The decontamination operation decreased the FNR, particularly for the SECM samples with a maternal contamination ratio between 25% and 50%. For these samples, both the decontamination operation alone and the decontamination operation combining the predecontamination CNV threshold at 1.8&2.2 reduced the FNR from 50% (7/14, 95% CI: 24%–76%) in the raw data to 21% (3/14, 95% CI: 0%–43%) after decontamination; the FNR was 29% (4/14, 95% CI: 5%–52%) and 43% (6/14, 95% CI: 17%–69%) when the predecontamination CNV thresholds were combined at 1.7&2.3 and 1.6&2.4, respectively. For the SECM samples with maternal contamination ratios between 50% and 75%, the FNRs were 100% (7/7, 95% CI: 90%–100%), 71% (5/7, 95% CI: 38%–100%), 86% (6/7, 95% CI: 60%–100%), 100% (7/7, 95% CI: 90%–100%), and 100% (7/7, 95% CI: 90%–100%) for the raw data, decontamination alone, and decontamination combined with the predecontamination CNV thresholds of 1.8&2.2, 1.7&2.3 and 1.6&2.4, respectively (Fig. 4a).

The decontamination operation increased the overall FPRs. For the SECM samples with a maternal contamination ratio between 25% and 50%, the FPRs were 40% (2/5, 95% CI: 2%–83%) in all groups. For SECM samples with a maternal contamination ratio between 50% and 75%, incorporating predecontamination CNV thresholds helped reduce the FPR compared with decontamination alone. The FPRs were 17% (2/12, 95% CI: 0%–38%), 50% (6/12, 95% CI: 22%–78%), 25% (3/12, 95% CI: 0%–49%), 25% (3/12, 95% CI: 0%–49%), and 17% (2/12, 95% CI: 0%–38%) for the raw data, decontamination alone, and decontamination combined with the predecontamination CNV thresholds of 1.8&2.2, 1.7&2.3 and 1.6&2.4, respectively (Fig. 4b). Analysing the CNV status of SECM using only the predecontamination CNV thresholds yielded poor results (Supplementary Fig. S6b). The decontamination process, combined with predecontamination CNV thresholds of 1.8&2.2, also helped reduce the sex discordance rate (SDR) and improved the overall concordance rate (GCR) (Supplementary Fig. S6c).

**Fig. 5:**
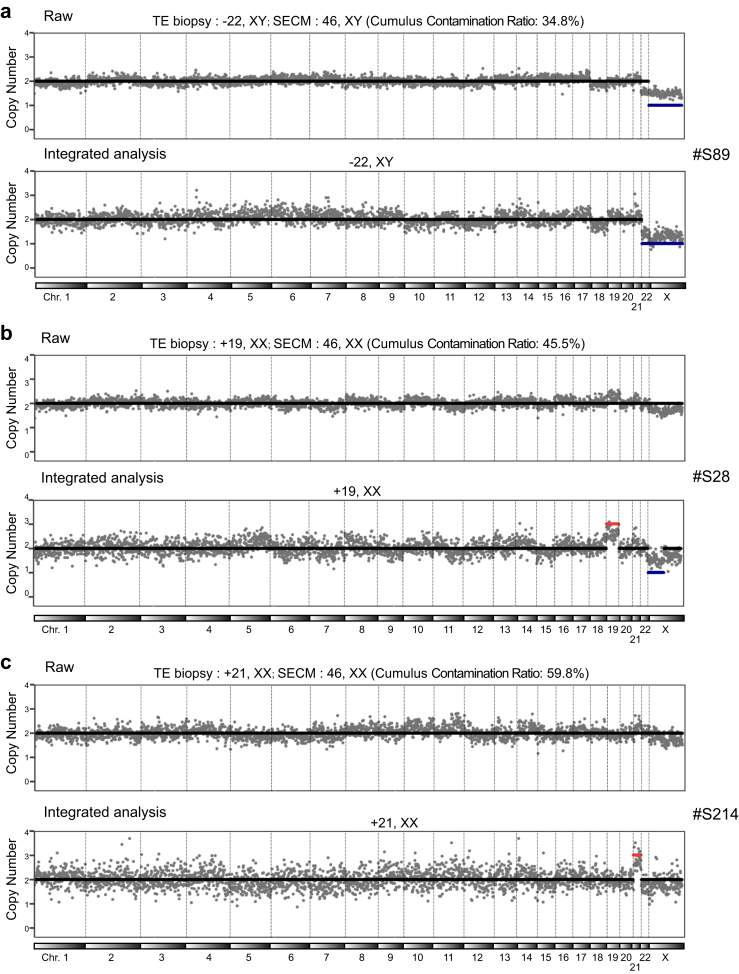
**CNVs of real SECMs with different proportions of cumulus cells before and after integrated analysis.** Original CNV and CNV after integrated analysis of real SECM and corresponding TE biopsy results. SECM with mild (a) to moderate (b) to high (c) levels of cumulus cell contamination.

Fig. 5 shows three examples. SECM sample #S89, the contamination rate was 34.8%, and the initial SECM result suggested a euploid status, whereas the TE biopsy result for the corresponding embryo was −22, XY. Following the decontamination process, the SECM result was corrected to −22, XY (Fig. 5a). For sample #S28 had a cumulus cell contamination ratio of 45.5%, with the raw SECM CN result being 46, XX; concurrently, the TE biopsy result for the corresponding embryo was +19, XX. After the decontamination operation, which included the predecontamination CNV threshold at 1.8&2.2, the SECM result was corrected to +19, XX, aligning with the TE biopsy result (Fig. 5b). In another case, sample #S214 presented a contamination ratio of 59.8%, with the raw SECM CN result indicating euploidy, whereas the TE biopsy result was +21, XX. After the decontamination process, the SECM result was correctly revised to +21, XX (Fig. 5c).

Together, the results demonstrated that decontamination can effectively reduce FNR and that incorporation of the predecontamination CNV threshold at 1.8&2.2 can effectively reduce the FPR in real SECM samples.

### Decontamination analysis for noncumulus cell contamination

We next investigated whether selecting hypomethylated reads could also reduce sperm DNA contamination in SECM, which should be an important source of nonembryonic contamination in the context of IVF. Compared with cumulus cells, sperm contain an even greater hypermethylated genome, with an average methylation level of 82% (Supplementary Fig. S7a). The distribution of CpG sites and methylated reads within the sperm reveals a unique pattern that sets it apart from those observed in SECMs, the ICM and the TE. Approximately 65% of the reads contained at least one CpG site that provided information for read-level methylation analysis. An average of 23%, 14%, 8%, and 20% of the reads contained one, two, three or more than three CpGs, respectively. We calculated the percentage value of methylated CpGs to all CpGs on each read. For sperm, on average, 20% of all the CpG-containing reads had a methylation value of 0%, 8% had a value between 1% and 99%, and 72% had a value of 100% (Supplementary Fig. S7b). Thus, if unmethylated reads are selected, the blastocyst DNA is expected to be enriched over the sperm DNA by a factor of approximately 2.5 [47% divided by 20%, where 47% and 20% refer to the average proportions of reads with a CpG methylation of 0 in the blastocyst-representative samples (which include the SECM with minor contamination, ICM, and TE), and the sperm samples, respectively].

We used euploid SECM samples that were free from maternal contamination (n = 23) for simulation analysis and synthesised data with varying proportions of contaminated sperm. The results revealed that when the sperm DNA proportion was 20%, 50%, 75%, and 90% of the total DNA, the decontamination algorithm with a precontamination threshold of 1.8&2.2 achieved sensitivities of 87%, 74%, 17%, and 0%, respectively, with specificities between 96% and 100% (Supplementary Fig. S7c and Table S1). Similarly, when incorporating the pre-contamination threshold of 1.8&2.2, it increased specificity while maintaining sensitivity to a certain extent, compared to decontamination alone.

**Fig. 6:**
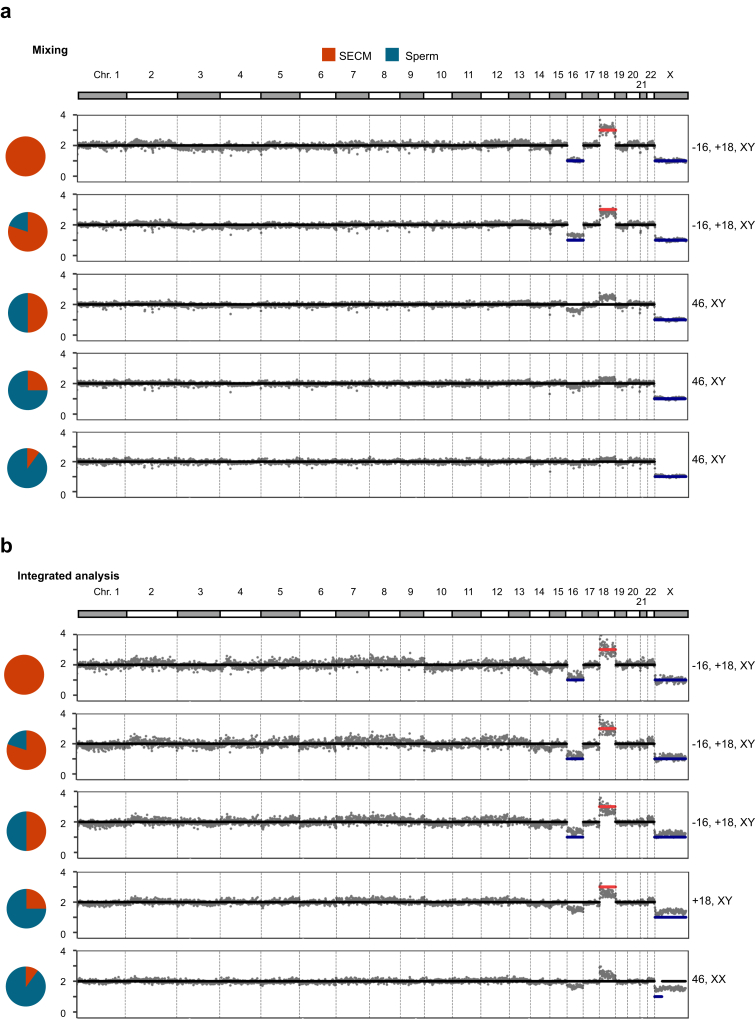
**CNVs of simulated SECMs with different proportions of sperm cells before and after integrated analysis.** (a) Raw CNVs of simulated samples with different proportions of sperm cells. The proportions of sperm cells were 20%, 50%, 75% and 90%, respectively. (b) CNV of simulated samples with different proportions of sperm cells after integrated analysis. The proportions of sperm cells were 20%, 50%, 75% and 90%, respectively. Among them, SECMs are completely uncontaminated culture medium samples.

For example, the data of an aneuploid SECM sample (−16, +18, XY) were mixed with the data of sperm cells in different proportions. With increasing proportions of sperm cells, the number of aneuploid chromosome signals increased to 2. When the proportion of sperm cell contamination was greater than 50%, no CNV was detected (Fig. 6a). After decontamination, the CN gain of chromosome 18 was restored in the group with a sperm proportion of 75%, and the CN loss of chromosome 16 was restored in the group with a 50% sperm proportion (Fig. 6b).

We also asked whether polar body cell contamination can be reduced by this approach.^10^ The methylation data of the MII oocyte were used as proxies for the polar body because of their similar DNA methylation patterns.^18^^,^^25^ The analysis revealed that 38% of all the CpG-containing reads in the MII oocyte methylation data were unmethylated, indicating that selecting unmethylated reads could achieve only a 1.2-fold enrichment of blastocyst DNA over polar body DNA (47% divided by 38%; Supplementary Fig. S7a and b). We performed simulation analysis by mixing the data of euploid SECM samples (n = 23) and MII oocytes at various ratios. The results showed that the decontamination algorithm, with a precontamination threshold of 1.8&2.2, achieved sensitivities of 83% (19/23), 52% (12/23), 9% (2/23), and 0% (0/23), for the data with polar body DNA proportions of 20%, 50%, 75%, and 90%, respectively (Supplementary Fig. S7c and S8). Interestingly, when the real clinical SECM samples were divided into two groups, those with polar body contamination ratios less than 25% and those with ratios between 25% and 50%, both groups exhibited a similarly reduced FNR (Supplementary Fig. S7d).

Together, the results indicate that decontamination treatment can effectively reduce sperm contamination and has some effectiveness in reducing polar body contamination.

## Discussion

In this study, we developed a DNA methylation-based computational method that efficiently reduced contamination from nonembryonic DNA and decreased the FNR of aneuploidy detection in SECM cfDNA. When cumulus cell contamination is present at a 50% ratio, it is estimated that the original CNV signal—where CN = 1 for a loss and CN = 3 for a gain, or simply 1&3—will be reduced by half, i.e., to CN = 1.5&2.5, potentially falling below the software's regular cutoff thresholds of 1.5&2.5. We demonstrated that by selecting unmethylated reads, blastocyst DNA can be enriched at a ratio of 4. Consequently, this computational operation is expected to increase the aneuploidy signal to 1.2&2.8. However, if the contamination ratio is as high as 75%, the aneuploidy signal will be reduced to 1.75 and 2.25, and the decontamination process is expected to only increase the signal to 1.43 and 2.57, which could still be challenging to detect. In line with this estimation, our findings showed that the decontamination procedure improved the sensitivity of aneuploidy detection at a 50% cumulus cell contamination ratio for both simulated and clinical SECM samples. However, its effectiveness decreased at a 75% contamination ratio.

We demonstrated that the inclusion of predecontamination CNV thresholds of 1.8&2.2 increased specificity without sacrificing sensitivity. As mentioned, at a cumulus cell contamination ratio of 50%, the predecontamination aneuploidy signal is reduced to 1.5&2.5; thus, a CNV threshold set at 1.8&2.2 should be capable of detecting the aneuploidy signal. Indeed, the FNR of the decontamination alone and the approach integrated with a predecontamination threshold of 1.8 and 2.2 showed similarly reduced FNR for the clinical SECM samples with cumulus cell contamination ratio between 25% and 50%. The impact of enhancing specificity is most evident in clinical samples, where the use of predecontamination CNV thresholds of 1.8&2.2 alone and decontamination alone increased the FPR, whereas the combination of the two resulted in an FPR comparable to that of the original data.

Recent studies have reported various strategies for optimising the sample collection process in SECM analysis, including optimising the timing of SECM collection, enhancing embryo rinsing techniques, and updating the culture media.^16^^,^^26^^,^^27^ A recent study revealed that denudation at both day 0 and day 3 post-ICSI can improve the sex ratio compared with that of routine sole day 0 denudation.^28^ By combining these physical operations with DNA methylation analysis and the computational decontamination method, it is feasible to address the cumulus cell contamination issue in SECM.

While ICSI is commonly used to address severe male factor infertility, traditional IVF is a common method for artificial insemination in nonmale factor infertile couples.^29^ In IVF, sperm that adhere to the zona pellucida may lead to paternal-originated contamination.^30^ Our results indicate that the hypomethylated read selection strategy can also improve aneuploidy detection in the situation of sperm contamination, which enriches the blastocyst DNA by a factor of approximately 2.5, thereby expanding its potential application in niPGT for IVF.

For limitations, this study was initially designed with a primary focus on removing cumulus cell contamination in SECM samples. As a result, the decontamination algorithm performs optimally with cumulus cells but has limited effectiveness in addressing polar body contamination. In the future, we aim to develop computational methods that specifically target the distinct characteristics of polar bodies to improve decontamination in this area. The investigation of sperm contamination is solely based on computer-simulated data. The strategy proposed in this study primarily considers DNA methylation characteristics to differentiate cell types, while other features are not incorporated. Moving forward, we intend to integrate various features to develop a more systematic and comprehensive computational approach. This is a proof-of-concept study designed to develop a computational algorithm for eliminating contaminated non-embryonic DNA from SECM. We only retrieved previously published data for our analysis; the total SECM sample size was 194, which was further augmented by creating computationally synthesised samples. Given the high degree of heterogeneity in SECM samples, the number of samples in certain contamination level subgroups in the clinical SECM analysis is relatively insufficient. Despite that we are able to establish the algorithm, future investigations will be necessary to expand the sample size to achieve more stable and robust results, as well as to facilitate clinical application of the algorithm.

Together, our study provides a novel computational approach for reducing the amount of contaminated non-embryonic DNA and improving the sensitivity of aneuploidy detection in SECM cfDNA methylation data. In combination with the use of the DNA methylation method for detecting SECM cfDNA, this approach shows great promise for applications in niPGT.

## Contributors

Conceptualization: Jie Qiao, Lu Wen, Yidong Chen.

Data curation: Yidong Chen, Lu Wen, Jin Huang.

Formal analysis: Yidong Chen, Lu Wen.

Funding acquisition: Yidong Chen, Jin Huang.

Investigation: Yidong Chen, Lu Wen.

Methodology: Yidong Chen, Lu Wen, Fuchou Tang.

Project administration: Jie Qiao, Lu Wen, Yidong Chen.

Resources: Jie Qiao, Lu Wen, Yidong Chen, Fuchou Tang.

Software: Lu Wen, Yidong Chen.

Supervision: Jie Qiao, Lu Wen, Yidong Chen.

Validation: Jie Qiao, Lu Wen, Yidong Chen, Fuchou Tang.

Visualization: Yidong Chen, Lu Wen.

Writing—original draft: Yidong Chen, Lu Wen.

Writing—review & editing: Jie Qiao, Fuchou Tang, Jin Huang.

L.W., Y.C. and J.Q. conceived the project. Y.C. conducted all the studies. Y.C. performed the bioinformatics analysis. Y.C. and L.W. wrote the manuscript, with contributions from all of the authors. All authors read and approved the final version of the manuscript.

## Data sharing statement

The data are available upon request from the authors. All the sequencing data used in this study were obtained from our team's previous research,^10^ and the data have been deposited in the National Genomics Data Centre (https://bigd.big.ac.cn/gsa/), with accession number (BioProject PRJCA003453) HRA000332. Our source codes are publicly available at https://github.com/jasminexiao/niPGT.

## Declaration of interests

The authors declare that they have no competing financial interests.

## Appendix ASupplementary data

Supplementary Table S2

Supplementary Figures Caption

Supplementary Tables Caption
