# Supplementary figures and images for: A computational DNA methylation method to remove contaminated DNA from spent embryo culture medium for noninvasive preimplantation genetic testing
Source: eBioMedicine. 2025 Mar 29;114:105669. doi: 10.1016/j.ebiom.2025.105669 (PMC11994334; doi:10.1016/j.ebiom.2025.105669)

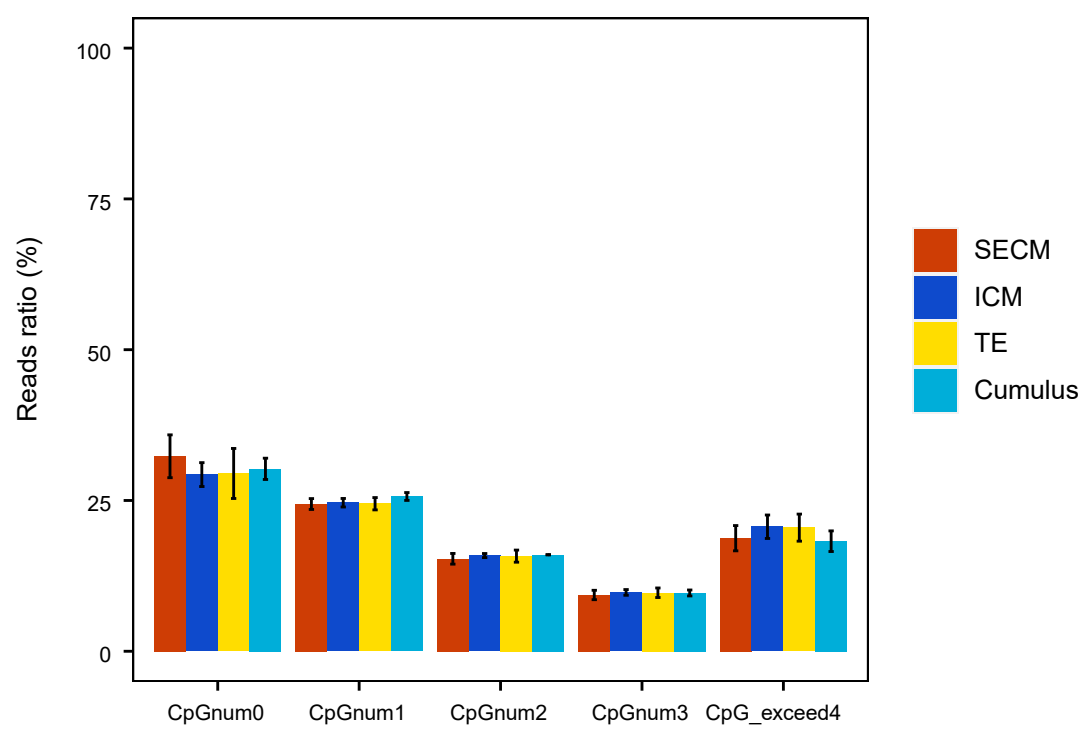

Supplement: Supplementary Fig. S1 [file mmc1.pdf]

a

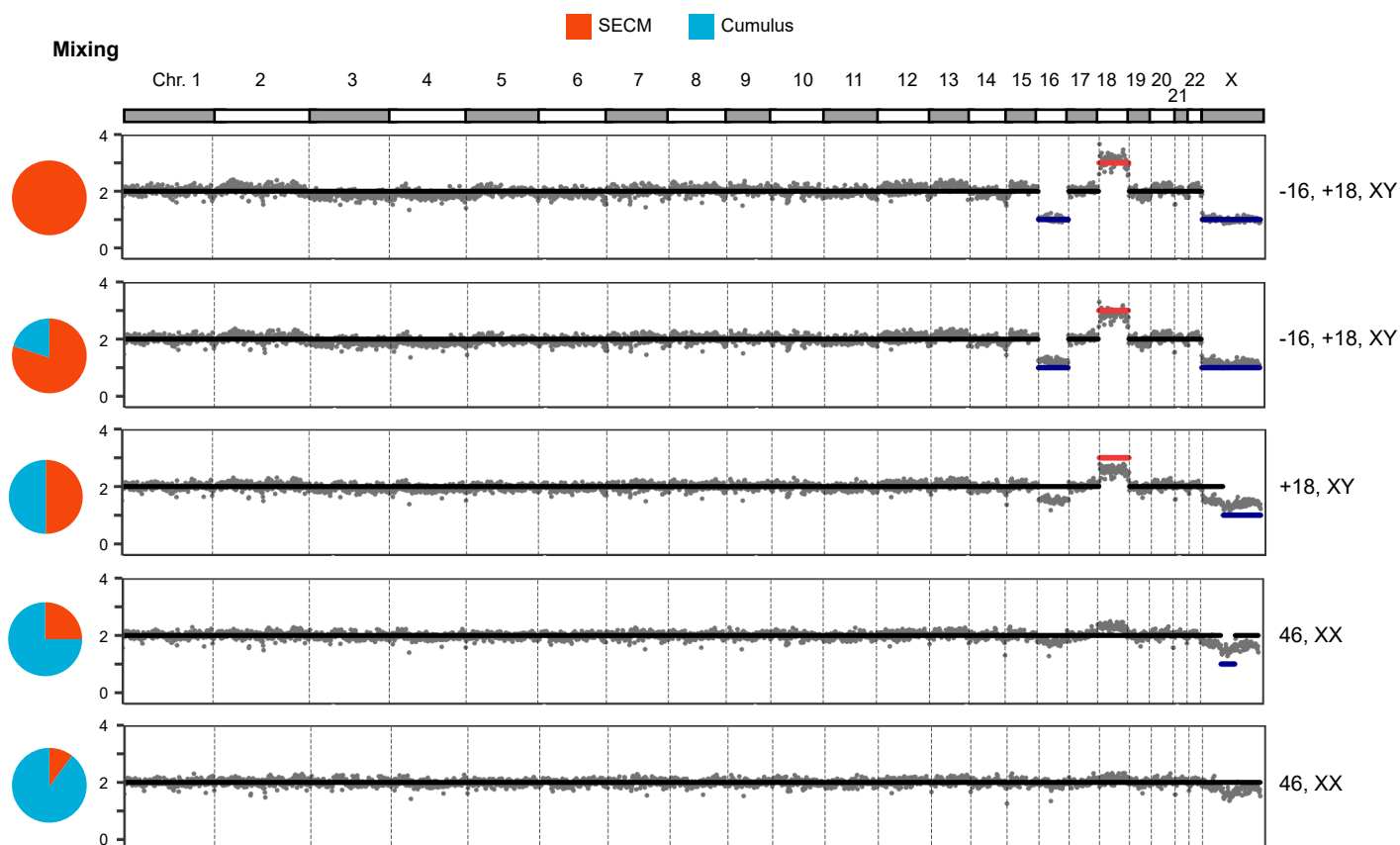

b

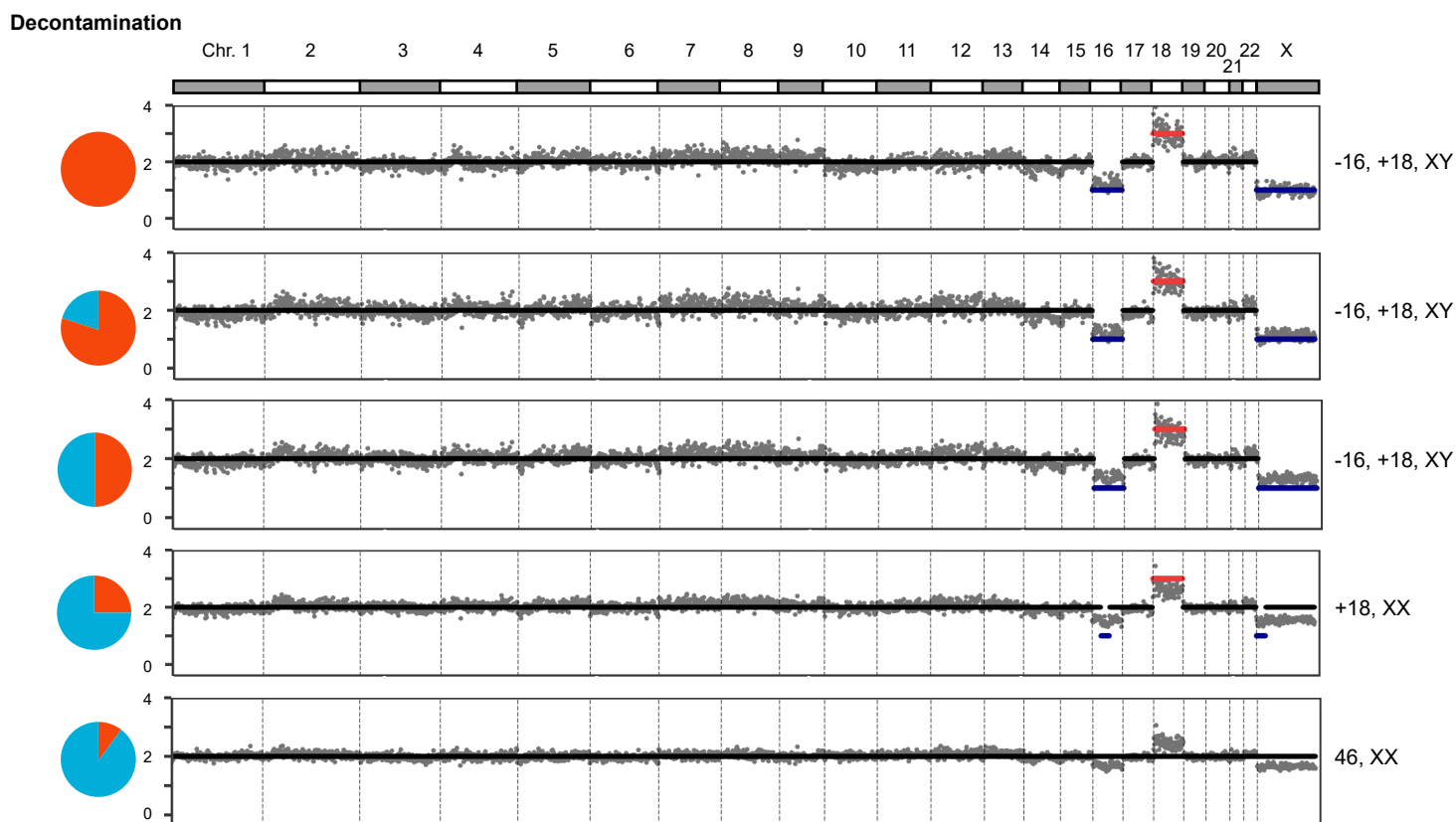

Supplement: Supplementary Fig. S2 [file mmc2.pdf]

a

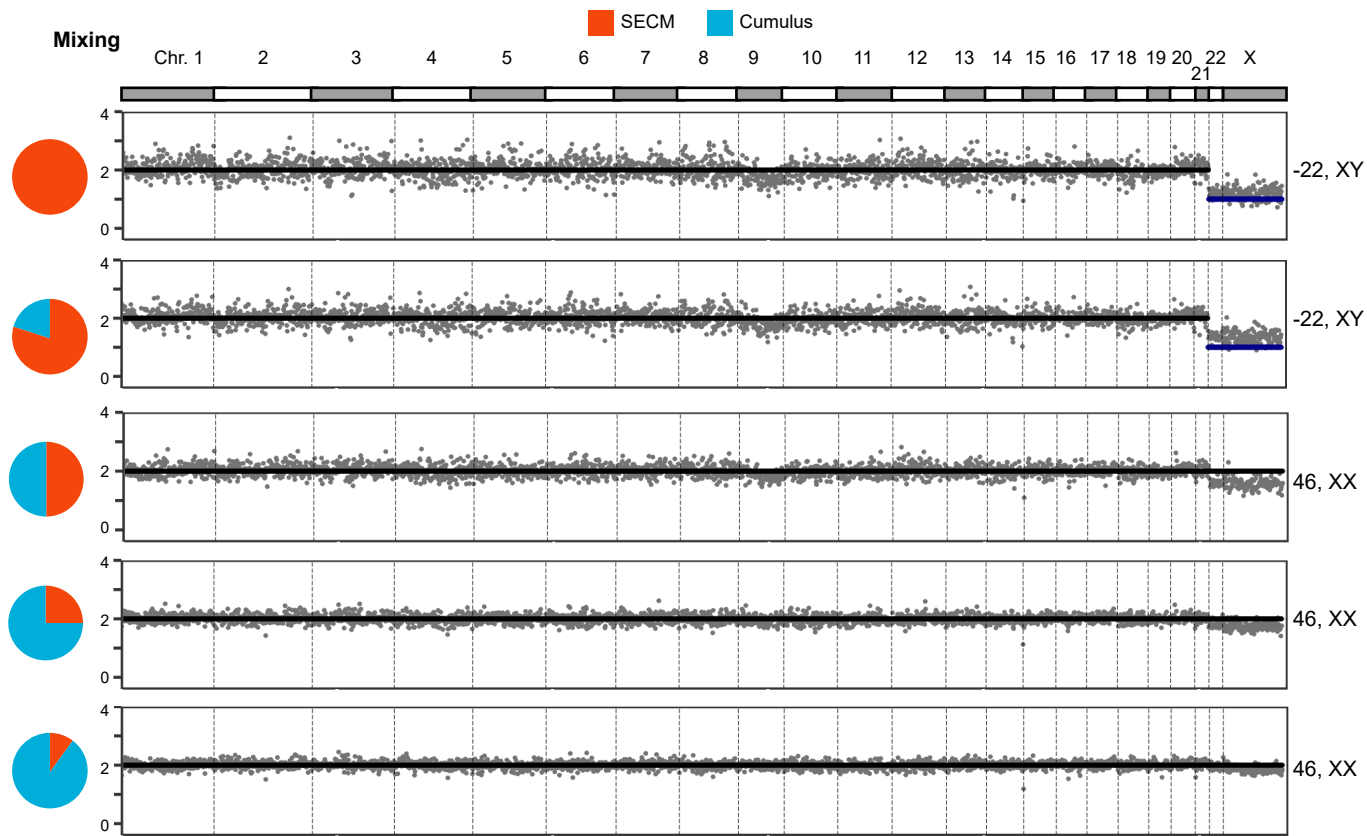

b

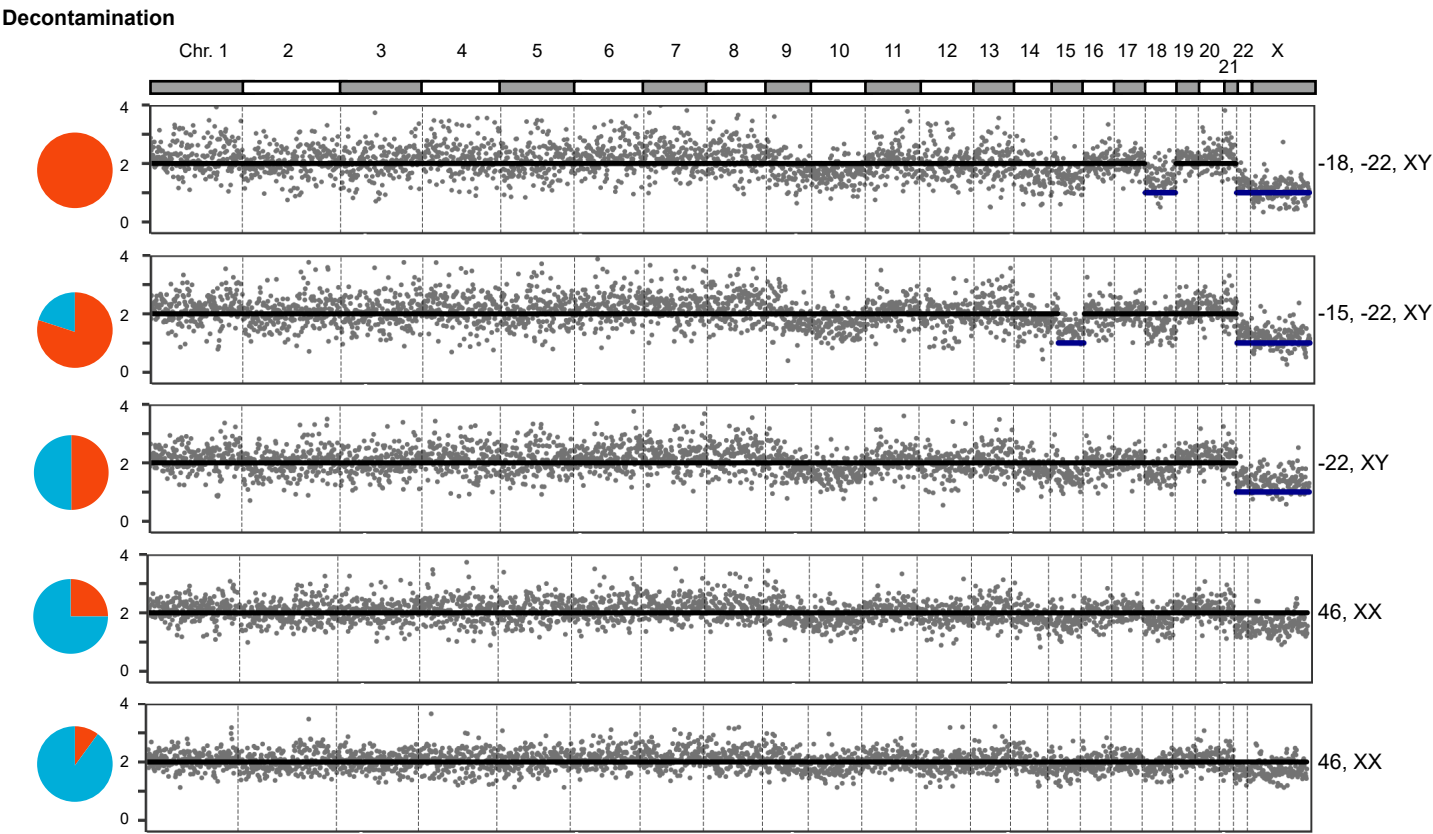

Supplement: Supplementary Fig. S3 [file mmc3.pdf]

a

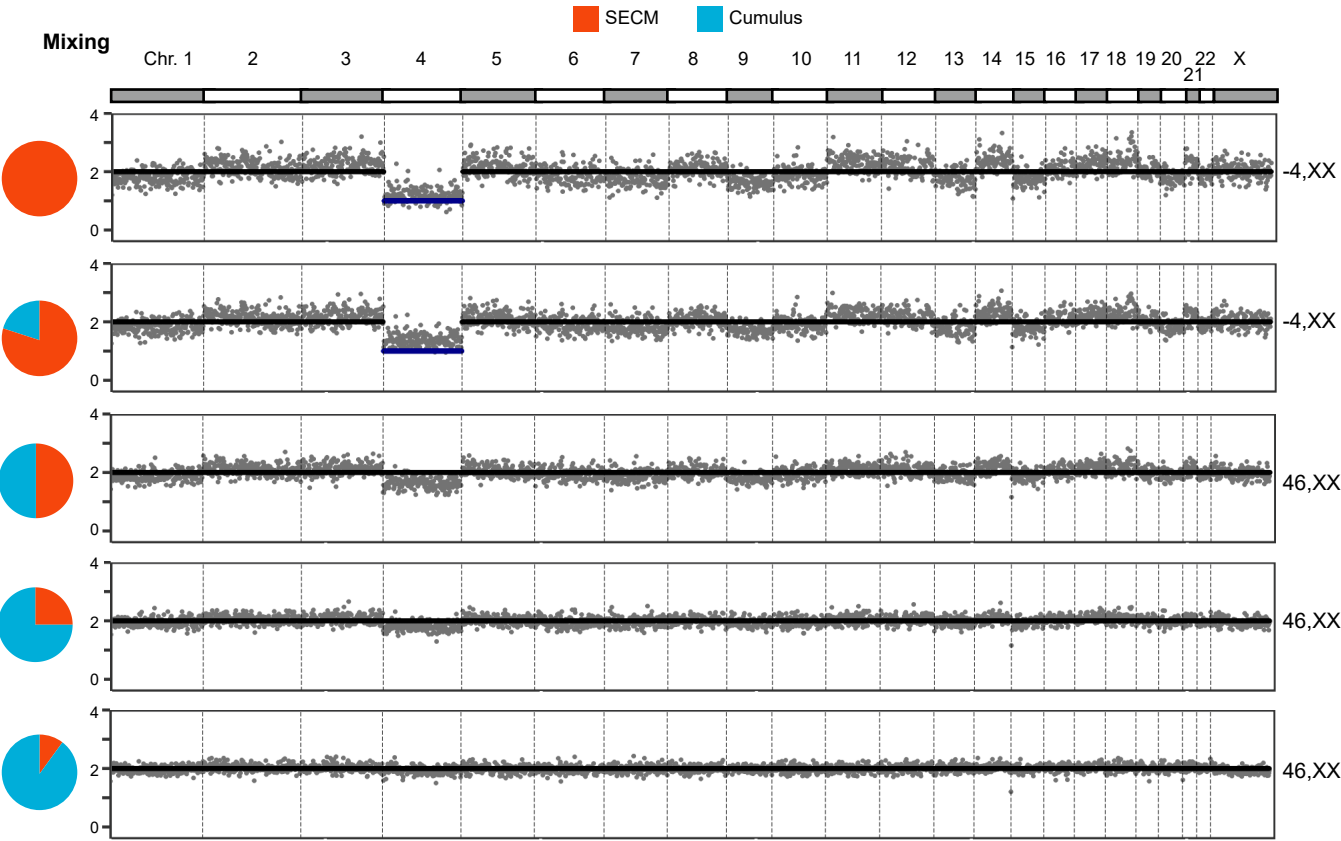

b

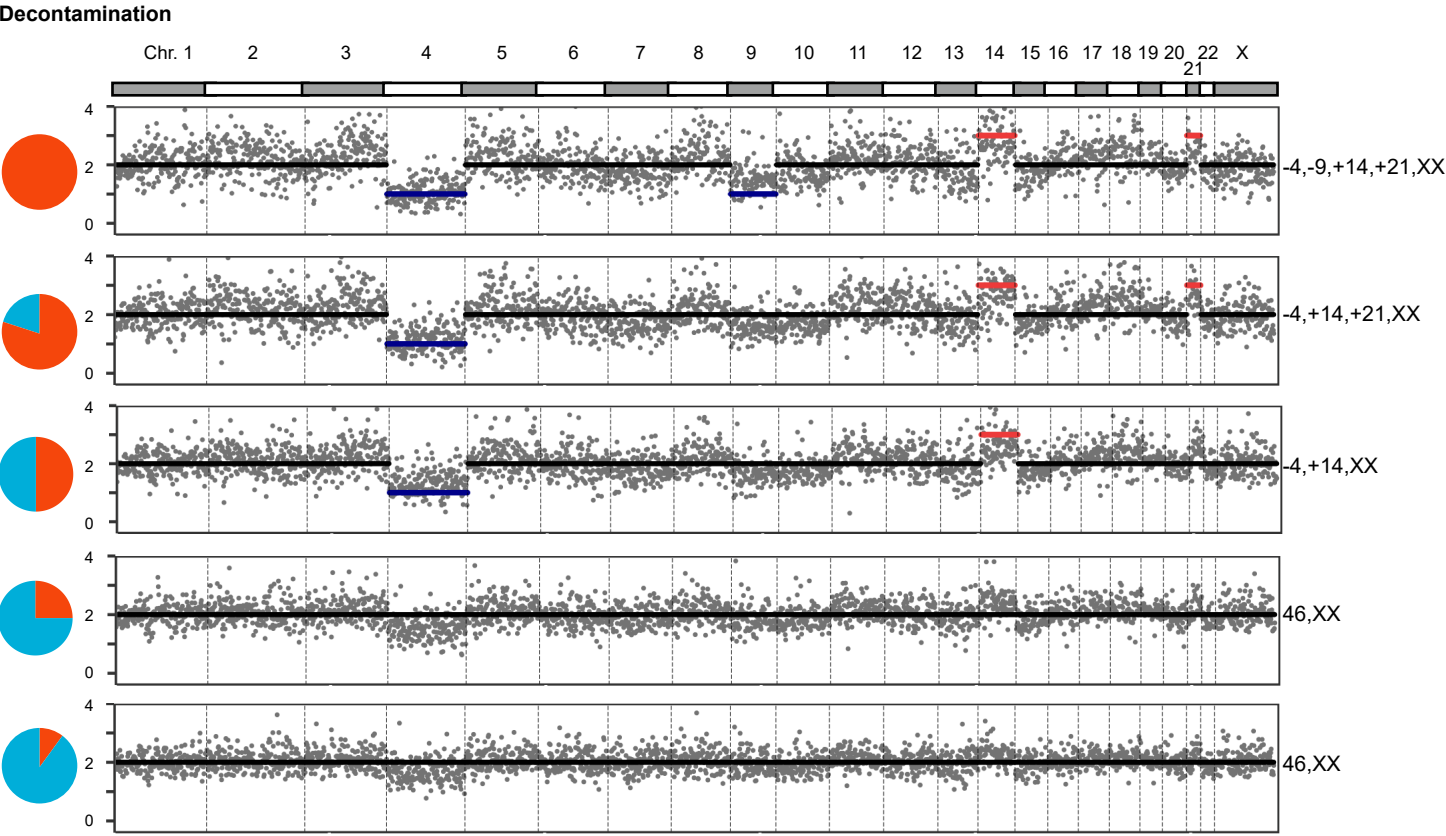

Supplement: Supplementary Fig. S4 [file mmc4.pdf]

a

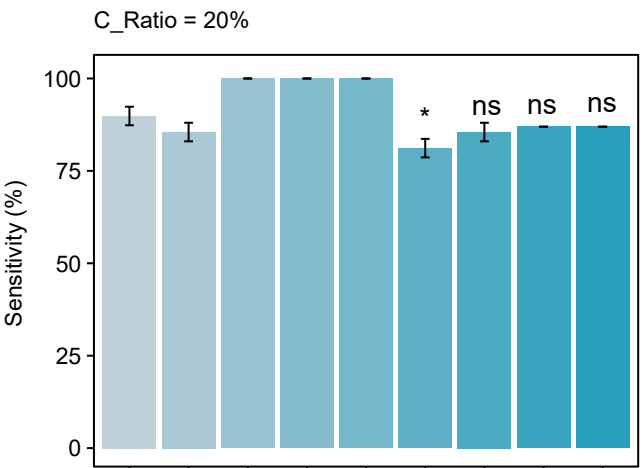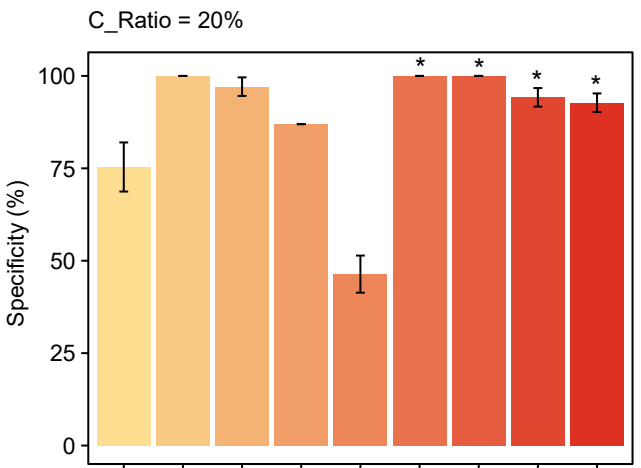

b

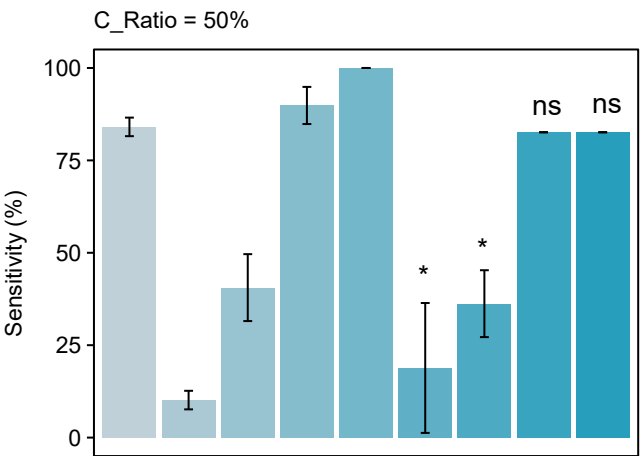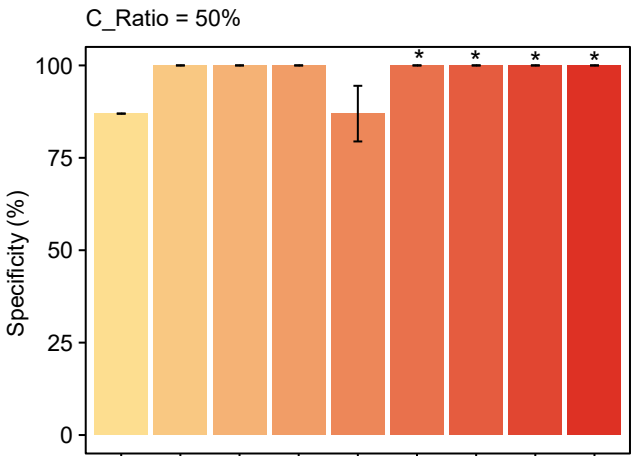

c

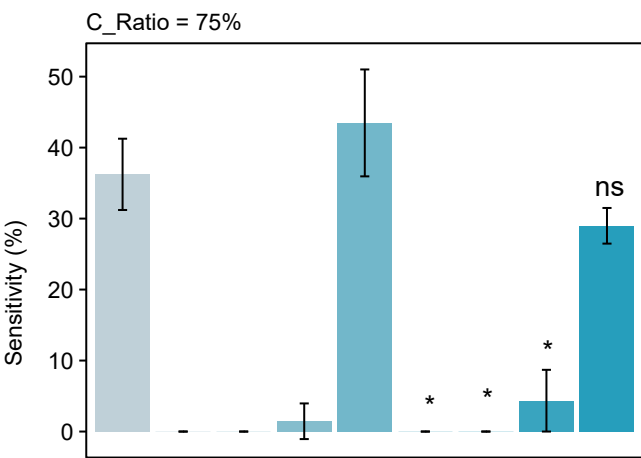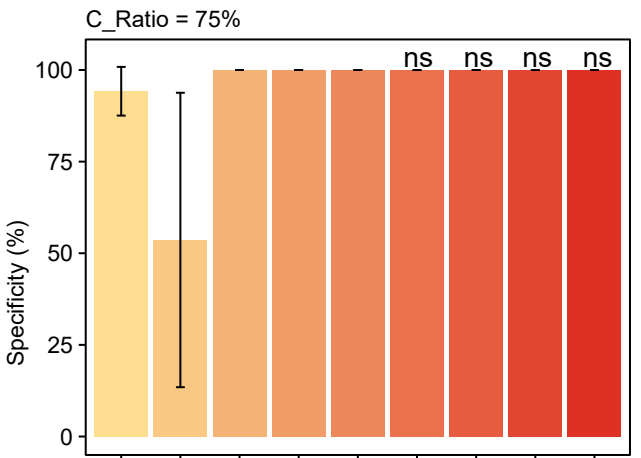

d

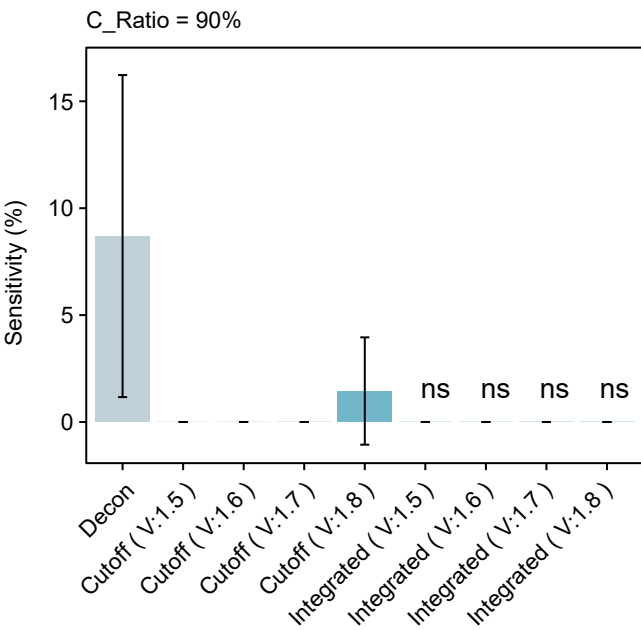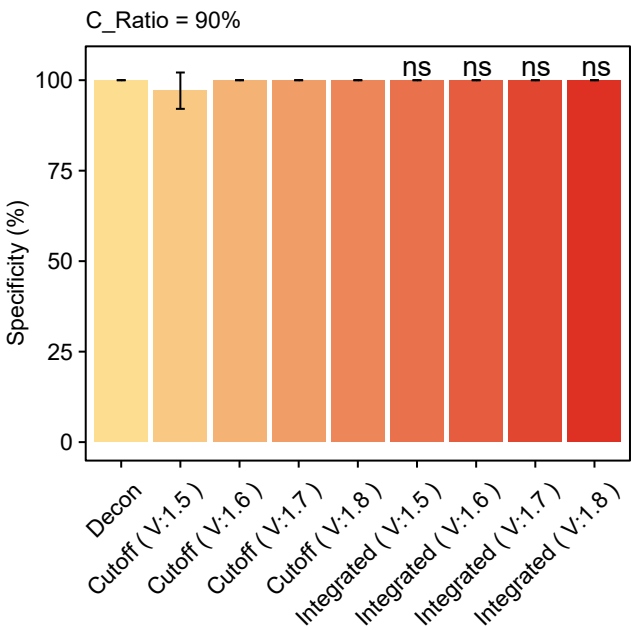

Supplement: Supplementary Fig. S5 [file mmc5.pdf]

a

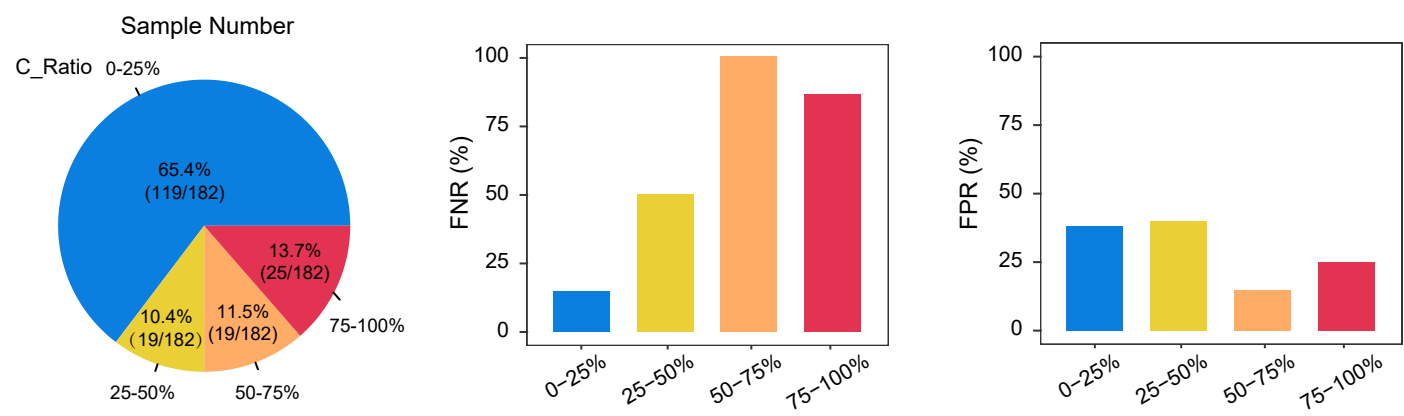

b

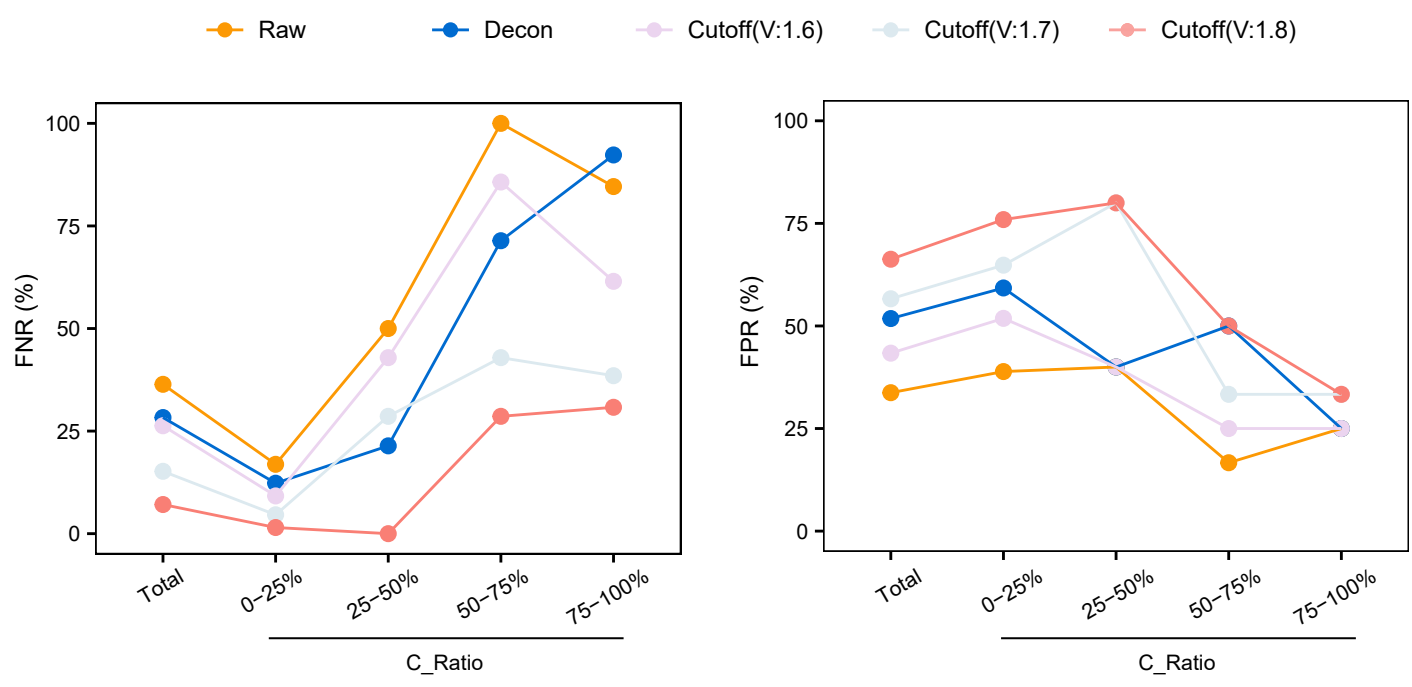

c

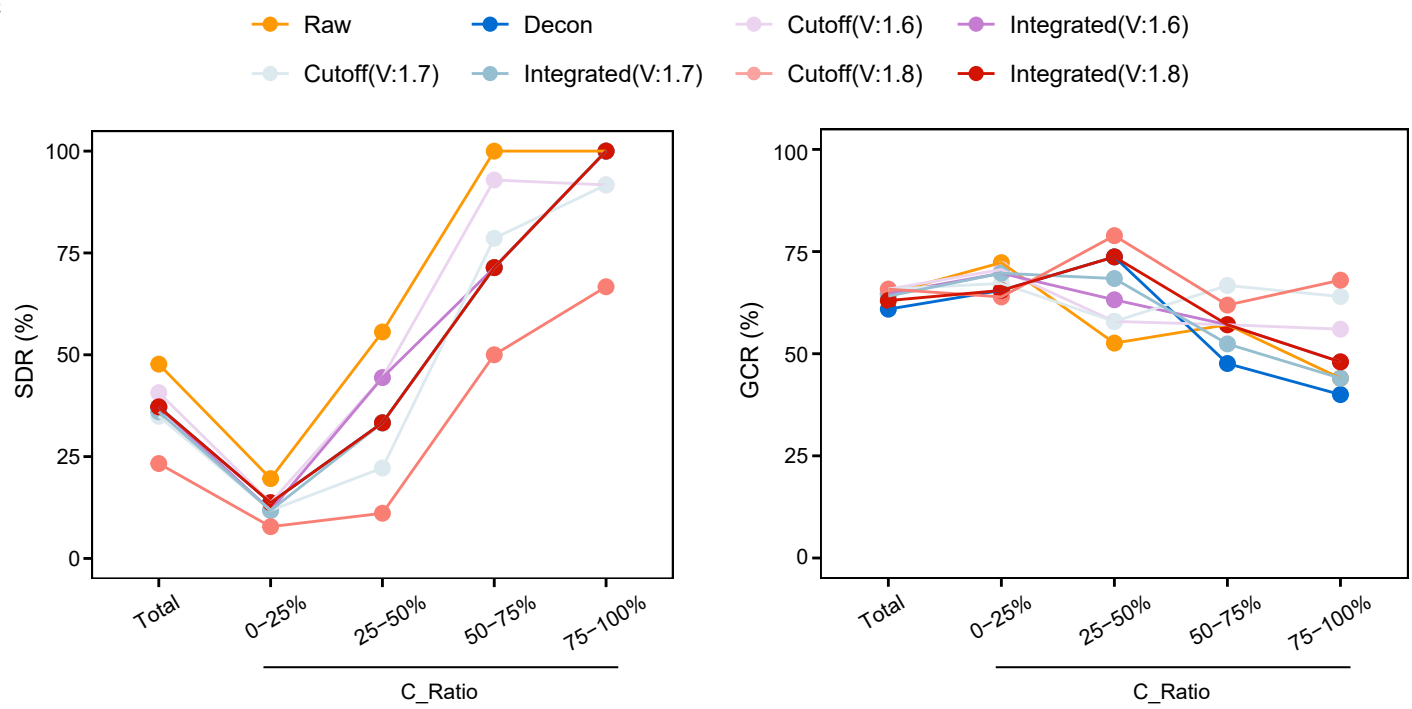

Supplement: Supplementary Fig. S6 [file mmc6.pdf]

a

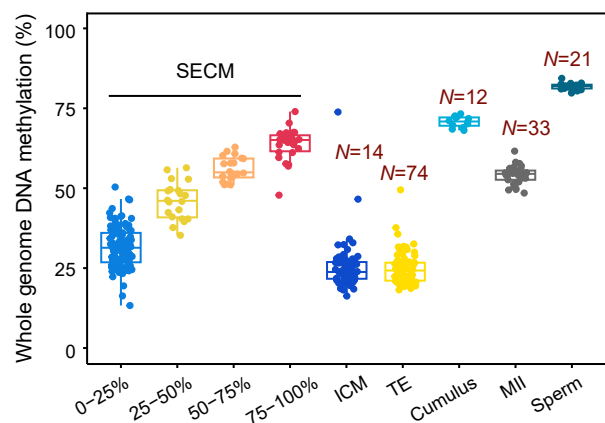

b

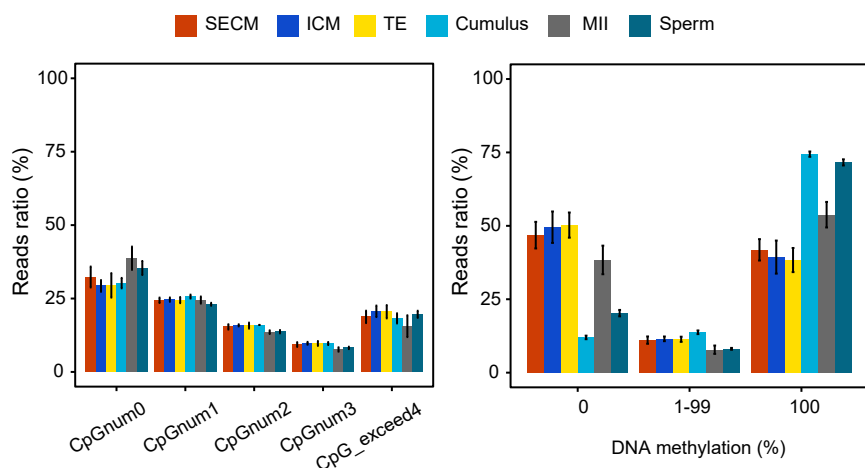

c

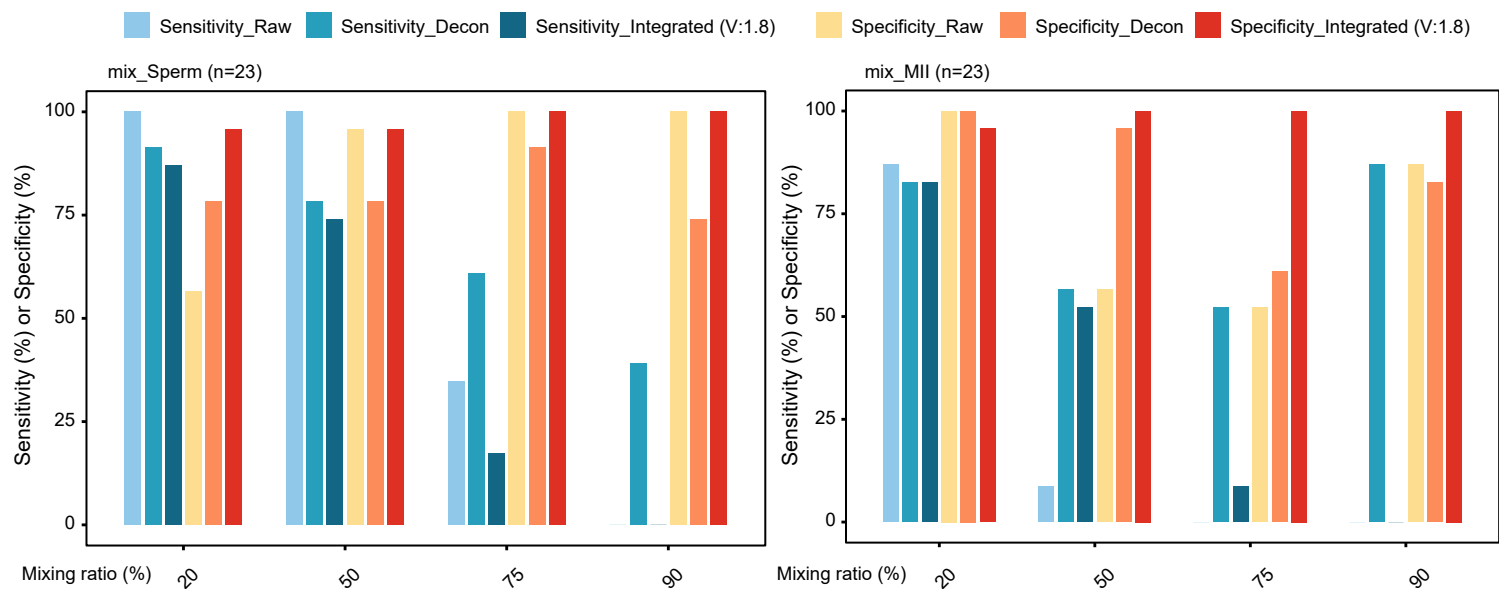

d

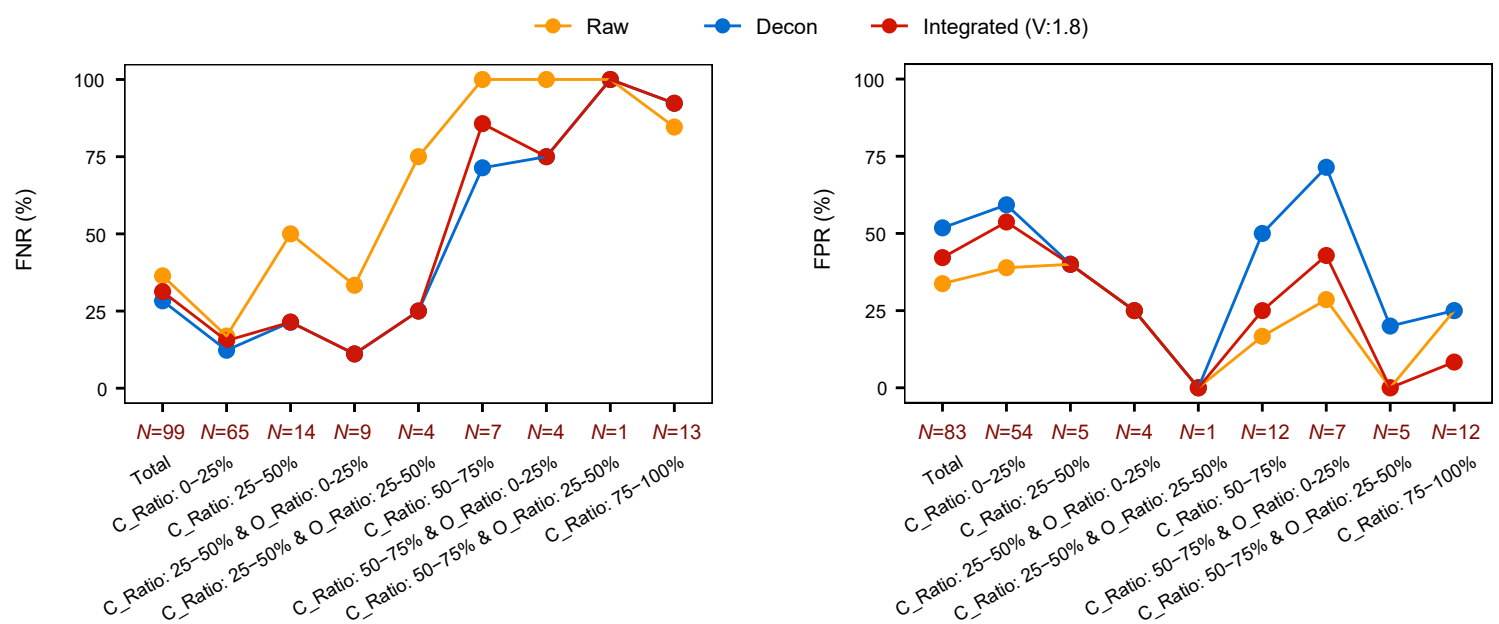

Supplement: Supplementary Fig. S7 [file mmc7.pdf]

a

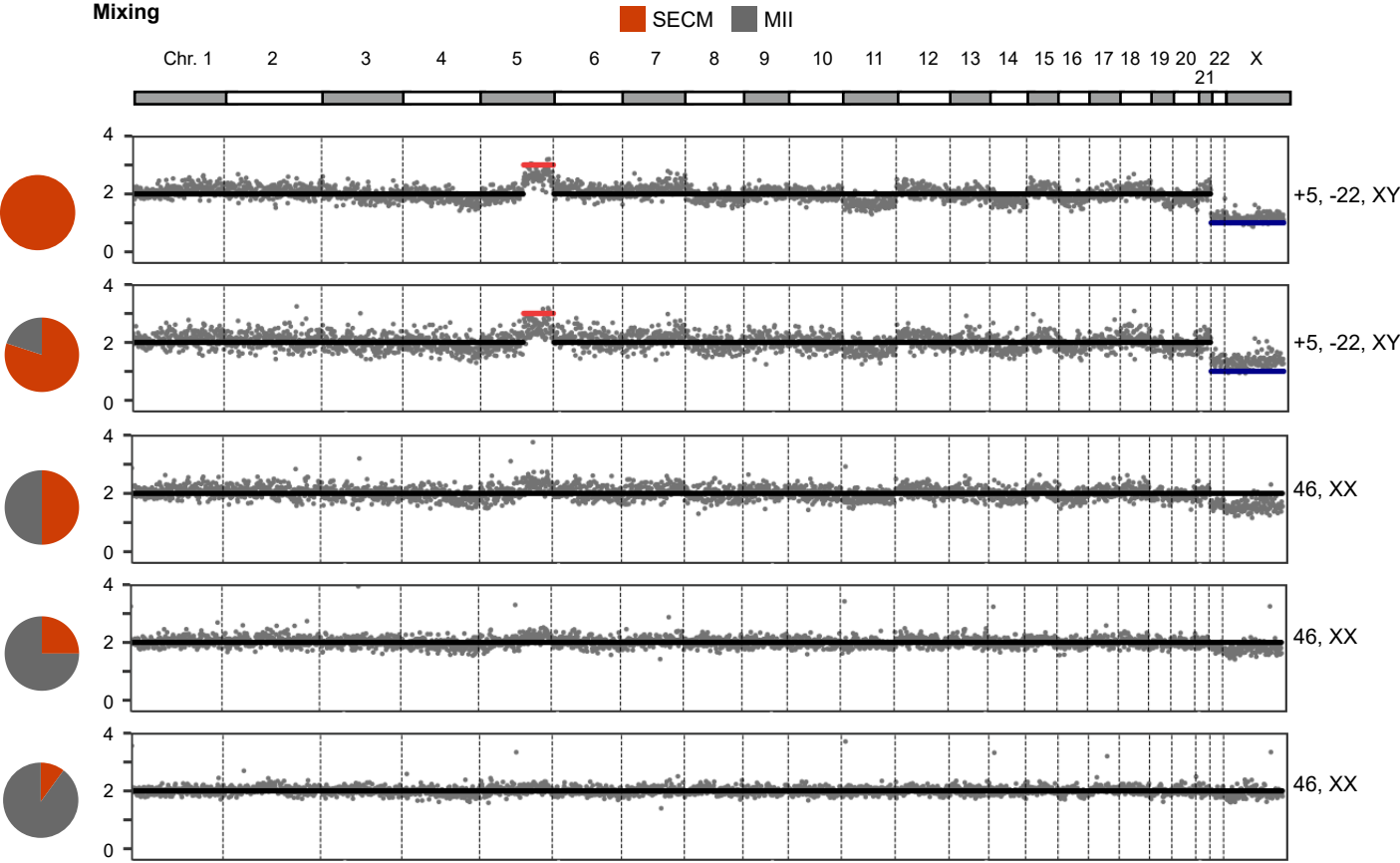

b

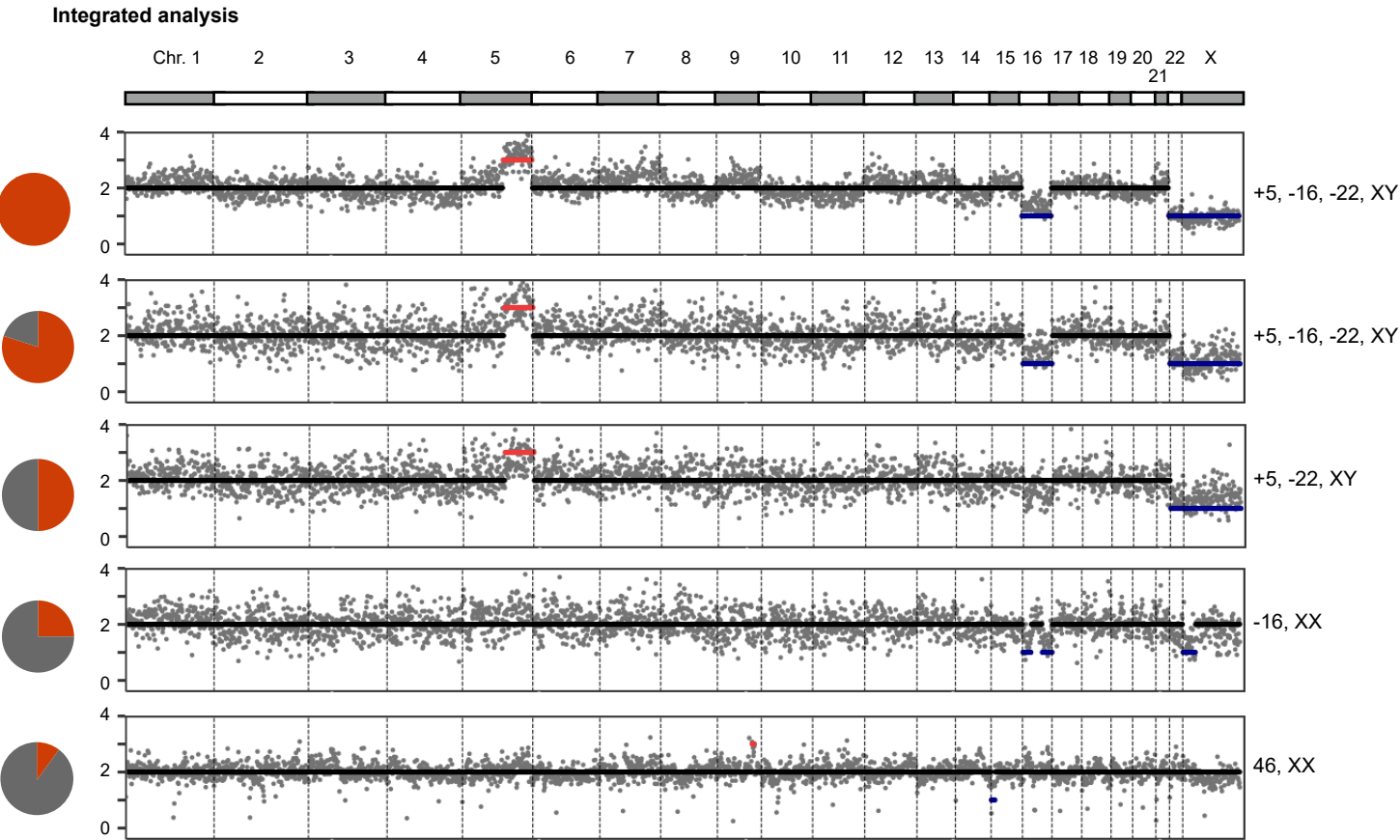

Supplement: Supplementary Fig. S8 [file mmc8.pdf]
