# Supplementary material for: A computational DNA methylation method to remove contaminated DNA from spent embryo culture medium for noninvasive preimplantation genetic testing
Source: eBioMedicine. 2025 Mar 29;114:105669. doi: 10.1016/j.ebiom.2025.105669 (PMC11994334; doi:10.1016/j.ebiom.2025.105669)
Supplement: Supplementary Figures Caption [file mmc11.docx]

**Supplementary Tables captions**

**Supplementary Table 1**

Simulated data CNV reporting threshold test

**Supplementary Table 2**

Sample information included quality control information, CNV before and after integrated analysis, maternal contamination, and sampling time.
