# Supplementary material for: A computational DNA methylation method to remove contaminated DNA from spent embryo culture medium for noninvasive preimplantation genetic testing
Source: eBioMedicine. 2025 Mar 29;114:105669. doi: 10.1016/j.ebiom.2025.105669 (PMC11994334; doi:10.1016/j.ebiom.2025.105669)
Supplement: Supplementary Tables Caption [file mmc12.docx]

**Supplemental Figure Legends**

**Suppl. Fig. 1. Distribution of the number of CpGs in each read of the SECM, ICM, TE and cumulus cells.** The X-axis represents the number of CpGs in each read segment. The Y-axis represents the ratio of read segments under the corresponding conditions. Among them, SECMs are completely uncontaminated culture medium samples.

**Suppl. Fig. 2. CNVs of other simulated SECMs with different proportions of cumulus cells before and after decontamination.** (a) Raw CNVs of simulated samples with different proportions of cumulus cells. The proportions of cumulus cells were 20%, 50%, 75% and 90%, respectively. (b) CNV of simulated samples with different proportions of cumulus cells after decontamination. The proportions of cumulus cells were 20%, 50%, 75% and 90%, respectively. Among them, SECMs are completely uncontaminated culture medium samples**.**

**Suppl. Fig. 3. CNVs of other simulated SECMs with different proportions of cumulus cells before and after decontamination.** (a) Raw CNVs of simulated samples with different proportions of cumulus cells. The proportions of cumulus cells were 20%, 50%, 75% and 90%, respectively. (b) CNV of simulated samples with different proportions of cumulus cells after decontamination. The proportions of cumulus cells were 20%, 50%, 75% and 90%, respectively. Among them, SECMs are completely uncontaminated culture medium samples.

**Suppl. Fig. 4. CNVs of other simulated SECMs with different proportions of cumulus cells before and after decontamination.** (a) Raw CNVs of simulated samples with different proportions of cumulus cells. The proportions of cumulus cells were 20%, 50%, 75% and 90%, respectively. (b) CNV of simulated samples with different proportions of cumulus cells after decontamination. The proportions of cumulus cells were 20%, 50%, 75% and 90%, respectively. Among them, SECMs are completely uncontaminated culture medium samples.

**Suppl. Fig. 5. Sensitivity and specificity under different reporting thresholds in various cumulus cell contamination samples.** Sensitivity and specificity under different reporting thresholds in various cumulus cell contamination samples, with the addition of 95% confidence intervals. A t-test was performed for each reporting threshold group to assess statistical significance compared to the decontamination alone group. The p-value obtained were as follows: 0.013, 0.1, 0.18, 0.18; 0.023, 0.023, 0.027, 0.033; 0.022, 0.008, 0.42, 0.42; 0.047, 0.047, 0.047, 0.047; 0.0063, 0.0063, 0.0012, 0.11; 0.27, 0.27, 0. 27, 0. 27; 0.18, 0. 18, 0. 18, 0. 18 (paired t-test). A p-value threshold of 0.05 was used to determine statistical significance. The analysis included a sample size of n = 23 with three biological replicates. The proportions of cumulus cells were 20% (a), 50% (b), 75% (c) and 90% (d), respectively.

**Suppl. Fig. 6. Formulation of decontamination strategy in real SECM.** (a) Pie chart and bar plots depicting the number of samples, FNR and FPR for different degrees of cumulus contamination. The pollution ratio groups included 0–25%, 25–50%, 50–75%, and 75–100% groups. (b) Line chart showing the FNR and FPR under different decontamination strategies. (c) Line chart showing the sex rate and global consistency rate under different decontamination strategies.

**Suppl. Fig. 7. Performance effects of decontamination strategies in noncumulus cell contamination.** (a) Box-and-whisker plot showing the whole-genome DNA methylation levels of SECM, TE, ICM, cumulus cells, MII oocytes and sperm cells. (b) Left: The X-axis represents the number of CpGs in each read segment. The Y-axis represents the ratio of read segments under the corresponding conditions. Among them, SECMs are completely uncontaminated culture medium samples. Right: The X-axis represents the average DNA methylation level on each read segment. The Y-axis represents the ratio of read segments under the corresponding conditions. Among them, SECMs are completely uncontaminated culture medium samples. (c) Sensitivity and specificity of different decontamination strategies in different sperm cell and MII oocyte contamination samples. (d) FNR and FPR between different groups of SECM**.**

**Suppl. Fig. 8. CNVs of simulated SECM with different proportions of MII oocytes before and after integrated analysis.** (a) Raw CNVs of simulated samples with different proportions of MII oocytes. The proportions of MII oocytes were 20%, 50%, 75% and 90%, respectively. (b) CNV of simulated samples with different proportions of MII oocytes after integrated analysis. The proportions of sperm cells were 20%, 50%, 75% and 90%, respectively. Among them, SECMs are completely uncontaminated culture medium samples.
